# Genome-wide Association for Major Depression Through Age at Onset Stratification: Major Depressive Disorder Working Group of the Psychiatric Genomics Consortium^☆^

**DOI:** 10.1016/j.biopsych.2016.05.010

**Published:** 2017-02-15

**Authors:** Robert A. Power, Katherine E. Tansey, Henriette Nørmølle Buttenschøn, Sarah Cohen-Woods, Tim Bigdeli, Lynsey S. Hall, Zoltán Kutalik, S. Hong Lee, Stephan Ripke, Stacy Steinberg, Alexander Teumer, Alexander Viktorin, Naomi R. Wray, Volker Arolt, Bernard T. Baune, Dorret I. Boomsma, Anders D. Børglum, Enda M. Byrne, Enrique Castelao, Nick Craddock, Ian W. Craig, Udo Dannlowski, Ian J. Deary, Franziska Degenhardt, Andreas J. Forstner, Scott D. Gordon, Hans J. Grabe, Jakob Grove, Steven P. Hamilton, Caroline Hayward, Andrew C. Heath, Lynne J. Hocking, Georg Homuth, Jouke J. Hottenga, Stefan Kloiber, Jesper Krogh, Mikael Landén, Maren Lang, Douglas F. Levinson, Paul Lichtenstein, Susanne Lucae, Donald J. MacIntyre, Pamela Madden, Patrik K.E. Magnusson, Nicholas G. Martin, Andrew M. McIntosh, Christel M. Middeldorp, Yuri Milaneschi, Grant W. Montgomery, Ole Mors, Bertram Müller-Myhsok, Dale R. Nyholt, Hogni Oskarsson, Michael J. Owen, Sandosh Padmanabhan, Brenda W.J.H. Penninx, Michele L. Pergadia, David J. Porteous, James B. Potash, Martin Preisig, Margarita Rivera, Jianxin Shi, Stanley I. Shyn, Engilbert Sigurdsson, Johannes H. Smit, Blair H. Smith, Hreinn Stefansson, Kari Stefansson, Jana Strohmaier, Patrick F. Sullivan, Pippa Thomson, Thorgeir E. Thorgeirsson, Sandra Van der Auwera, Myrna M. Weissman, CONVERGE Consortium, CARDIoGRAM Consortium, GERAD1 Consortium, Gerome Breen, Cathryn M. Lewis

**Affiliations:** a Institute of Psychiatry, Psychology, and Neuroscience, King׳s College London, London; b MRC Centre for Neuropsychiatric Genetics and Genomics, Institute of Psychological Medicine and Clinical Neurosciences, School of Medicine, Cardiff University, Cardiff, United Kingdom; c Lundbeck Foundation Initiative for Integrative Psychiatric Research, iPSYCH, Aarhus University, Aarhus, Denmark; d Translational Neuropsychiatry Unit, Department of Clinical Medicine, Aarhus University, Aarhus, Denmark; e Discipline of Psychiatry, School of Medicine, University of Adelaide, Adelaide, Australia; f Department of Psychiatry, Virginia Institute for Psychiatric and Behavioral Genetics, Virginia Commonwealth University, Richmond, Virginia; g Division of Psychiatry, University of Edinburgh, Edinburgh, United Kingdom; h Institute of Social and Preventive Medicine, Centre Hospitalier Universitaire Vaudois, Lausanne, Switzerland; i Queensland Brain Institute, The University of Queensland, Brisbane, Queensland; j School of Environmental and Rural Science, University of New England, Armidale, New South Wales, Australia; k Stanley Center for Psychiatric Research, Broad Institute of Massachusetts Institute of Technology and Harvard, Cambridge; l Analytic and Translational Genetics Unit, Massachusetts General Hospital and Harvard Medical School, Boston, Massachusetts; m Department of Psychiatry and Psychotherapy, Charité, Campus Mitte, Berlin, Germany; n deCODE Genetics, Reykjavik, Iceland; o Institute for Community Medicine, University Medicine Greifswald, Greifswald, Germany; p Department of Medical Epidemiology and Biostatistics, Karolinska Institutet, Stockholm, Sweden; q Department of Psychiatry and Psychotherapy, University of Muenster, Muenster, Germany; r Department of Biological Psychology, EMGO Institute for Health and Care Research and Neuroscience Campus Amsterdam, Vrije Universiteit, Amsterdam, The Netherlands; s Department of Biomedicine and Centre for Integrative Sequencing, iSEQ, Aarhus University, Aarhus, Denmark; t Department of Psychiatry, Lausanne University Hospital, Lausanne, Switzerland; u Department of Psychiatry, University of Marburg, Marburg, Germany; v Centre for Cognitive Ageing and Cognitive Epidemiology, Edinburgh, United Kingdom; w Department of Psychology, University of Edinburgh, Edinburgh, United Kingdom; x Institute of Human Genetics; Bonn, Germany; y Department of Genomics, Life and Brain Center, University of Bonn, Bonn, Germany; z QIMR Berghofer Medical Research Institute, Brisbane, Queensland, Australia; aa Department of Psychiatry and Psychotherapy, University Medicine Greifswald, Greifswald, Germany; ab Department of Psychiatry, Kaiser Permanente San Francisco Medical Center, San Francisco, California; ac Medical Genetics Section, Centre for Genomic and Experimental Medicine, Institute of Genetics and Molecular Medicine, University of Edinburgh, Edinburgh, United Kingdom; ad Department of Psychiatry, Washington University St. Louis, St. Louis, Missouri; ae Division of Applied Health Sciences, University of Aberdeen, Aberdeen, United Kingdom; af Interfaculty Institute for Genetics and Functional Genomics, University of Greifswald, Greifswald; ag Max Planck Institute of Psychiatry, Munich, Germany; ah Mental Health Center Copenhagen, Mental Health Services in Capital Region, University of Copenhagen, Copenhagen, Denmark; ai Institute of Neuroscience and Physiology, University of Gothenburg, Gothenburg, Sweden; aj Department of Genetic Epidemiology in Psychiatry, Central Institute of Mental Health, Medical Faculty Mannheim/Heidelberg University, Mannheim, Germany; ak Department of Psychiatry and Behavioral Sciences, Stanford University, Stanford, California; al Department of Psychiatry, EMGO Institute for Health and Care Research and Neuroscience Campus Amsterdam, VU University Medical Center, Amsterdam, The Netherlands; am Psychosis Research Unit, Aarhus University Hospital, Risskov, Denmark; an Munich Cluster for Systems Neurology, Munich, Germany; ao Institute of Translational Medicine, University of Liverpool, Liverpool, United Kingdom; ap Institute of Health and Biomedical Innovation, Queensland University of Technology, Brisbane, Queensland, Australia; aq Therapeia, Reykjavik, Iceland; ar Institute of Cardiovascular and Medical Sciences, University of Glasgow, Glasgow, United Kingdom; as Charles E. Schmidt College of Medicine, Florida Atlantic University, Boca Raton, Florida; at Department of Psychiatry, University of Iowa Carver College of Medicine, Iowa City, Iowa; au CIBERSAM-Universidad de Granada e Instituto de Investigación Biosanitaria ibs, Hospitales Universitarios de Granada/Universidad de Granada, Granada, Spain; av Division of Cancer Epidemiology and Genetics, National Cancer Institute, National Institutes of Health, Bethesda, Maryland; aw Department of Psychiatry, Group Health, Seattle, Washington; ax University of Iceland; ay Department of Psychiatry, Landspitali University Hospital, Reykjavik, Iceland; az Division of Population Health Sciences, University of Dundee, Dundee, United Kingdom; ba Department of Genetics, University of North Carolina at Chapel Hill, Chapel Hill, North Carolina; bb College of Physicians and Surgeons and the Mailman School of Public Health, Columbia University and New York State Psychiatric Institute, New York, New York

**Keywords:** Age at onset, GWAS, Heterogeneity, Major depressive disorder, Polygenic scoring, Stratification

## Abstract

**Background:**

Major depressive disorder (MDD) is a disabling mood disorder, and despite a known heritable component, a large meta-analysis of genome-wide association studies revealed no replicable genetic risk variants. Given prior evidence of heterogeneity by age at onset in MDD, we tested whether genome-wide significant risk variants for MDD could be identified in cases subdivided by age at onset.

**Methods:**

Discovery case-control genome-wide association studies were performed where cases were stratified using increasing/decreasing age-at-onset cutoffs; significant single nucleotide polymorphisms were tested in nine independent replication samples, giving a total sample of 22,158 cases and 133,749 control subjects for subsetting. Polygenic score analysis was used to examine whether differences in shared genetic risk exists between earlier and adult-onset MDD with commonly comorbid disorders of schizophrenia, bipolar disorder, Alzheimer’s disease, and coronary artery disease.

**Results:**

We identified one replicated genome-wide significant locus associated with adult-onset (>27 years) MDD (rs7647854, odds ratio: 1.16, 95% confidence interval: 1.11–1.21, *p* = 5.2 × 10^-11^). Using polygenic score analyses, we show that earlier-onset MDD is genetically more similar to schizophrenia and bipolar disorder than adult-onset MDD.

**Conclusions:**

We demonstrate that using additional phenotype data previously collected by genetic studies to tackle phenotypic heterogeneity in MDD can successfully lead to the discovery of genetic risk factor despite reduced sample size. Furthermore, our results suggest that the genetic susceptibility to MDD differs between adult- and earlier-onset MDD, with earlier-onset cases having a greater genetic overlap with schizophrenia and bipolar disorder.

Major depressive disorder (MDD) is a highly prevalent and heterogeneous disorder (1). With most individuals experiencing recurrent episodes throughout life (2), MDD is now the second leading cause of disability worldwide (3). MDD is defined by low mood and energy, inability to experience enjoyment, changes to eating and sleep patterns, feelings of guilt or worthlessness, and suicidal thoughts (4). Along with excess mortality and increased risk of suicide (5), MDD is associated with worse clinical outcomes when comorbid with health problems such as cardiovascular disease and cancer (6, 7). Although the heritability is estimated at 31% to 42% (8), the causal variants remain elusive: a recent large mega-analysis with over 9000 MDD cases failed to identify any replicable associations (9), despite successes in similarly sized studies of schizophrenia (SCZ) and bipolar disorder (BPD) (10, 11). This lack of biological markers may be among the causes for the well-established underfunding of research into MDD relative to its economic and health burden (12) and the reported stigmatization of sufferers (13).

However, several differences exist between MDD and other psychiatric disorders in which replicable genetic associations have been identified, including higher prevalence, greater diagnostic uncertainty, lower heritability, and, crucially, increased heterogeneity. One known source of heterogeneity that may contribute substantially is age at onset (AAO). Onset can occur at any stage of life, yet many factors associated with MDD are either age specific or age restricted. These include biological events such as puberty, menopause, and dementia, and environmental risk factors including childhood maltreatment, childbirth, and divorce. Earlier onset is associated with increased risk in first-degree relatives and with higher heritability (14, 15, 16, 17, 18). Considerable differences in the transmission of early- versus late-onset MDD have also been reported (19, 20), with some studies suggesting the effects of novel genetic risk factors for MDD appearing later in life (21, 22).

Here we build on the previous mega-analysis of the Major Depressive Disorder Working Group of the Psychiatric Genomics Consortium (PGC-MDD), using AAO to stratify cases within a sample of 8920 cases and 9521 control subjects, with the goal of reducing heterogeneity. For each of the nine PGC-MDD samples (23, 24, 25, 26, 27, 28, 29, 30), cases were ordered by AAO within study and divided into eight groups (octiles). Genome-wide association analysis of cases in these octiles was performed systematically against control subjects for 1,235,109 autosomal single nucleotide polymorphisms (SNPs). We examined three analytic strategies: 1) genetic variants specific to early-onset MDD; 2) risk variants specific to late-onset MDD; and 3) restricting to the intermediate four octiles excluding the 25% of cases at either extreme of AAO to test for potential heterogeneity introduced from very early or very late onset. Significantly associated SNPs were taken forward for replication in nine studies comprising 13,238 cases and 124,230 control subjects. We also examined the differences between early- and late-onset MDD in their shared heritability with commonly comorbid disorders of schizophrenia, bipolar disorder, Alzheimer’s disease, and coronary artery disease to identify differences in genetic etiologies across onset groups.

## Methods and Materials

### Description of Samples

Full details of the studies that form the PGC-MDD are given in the supplementary materials of the original data analysis (9). Briefly, these nine MDD studies (23, 24, 25, 26, 27, 28, 29, 30) conducted genome-wide genotyping on individual subjects of European ancestry. Subjects were required to have diagnoses of DSM-IV lifetime MDD established using structured diagnostic instruments from direct interviews by trained interviewers or clinician-administered DSM-IV checklists. Two studies required recurrent MDD and one study required recurrent, early-onset MDD. Studies ascertained cases mostly from clinical sources, and control subjects were largely randomly selected from the population and screened for lifetime history of MDD. This led to a total of 9238 cases and 9521 control subjects with genotype information.

### AAO Phenotype

AAO was defined as the age at which individuals first had symptoms that met the criteria of MDD and was self-reported in all studies. Of the original 9238 cases included in the sample, 8920 (95.6%) had a reported AAO. Cases reporting an AAO older than the recorded age at interview were removed from the analysis (*n* = 17). Within each study, cases were ordered by AAO and then divided into octiles, giving approximately 1000 cases per octile. Octiles were defined within each study to account for differences in case ascertainment. We noted a wide range in AAO between studies ascertaining recurrent depression using the same instrument, indicating that the precise setting (study, clinic, country) was important; we therefore chose to order cases by AAO within each study, rather than across studies, or by absolute AAO cutoffs. This strategy will identify genetic variants that were specific to early or late onset, relative to the mean AAO of the recruited cases. For secondary analysis of sex-specific effects, octiles were additionally defined within only male and female subjects for analysis of sex-specific effects, and for recurrent depression that has a higher heritability. These octiles will be referred to as O1 to O8, with O1 representing the earliest onset octile and O8 representing the latest onset octile. The GenRED (Genetics of Recurrent Early-Onset Depression) study only recruited MDD cases with an AAO below 31 years (25). We compared the distribution of AAO in GenRED with other similar studies (STAR*D [Sequenced Treatment Alternatives to Relieve Depression], RADIANT-UK) and estimated that GenRED recruited the youngest cases (62% of all possible MDD cases), with older cases absent from the study (Supplemental Figure S1). GenRED cases were ordered by AAO and assigned to the appropriate octiles O1 to O5, with no cases present in O6 to O8.

### Quality Control

Genotyping was described in the supplementary materials in the original analysis (11). All samples were genotyped with SNP arrays of at least 200,000 SNPs. SNPs were removed for missingness >0.02, case-control difference in SNP missingness >0.02, SNP frequency difference from HapMap3 (phase three of the International HapMap Project) >0.15, or deviation from Hardy–Weinberg equilibrium in control subjects (exact *p* < 1.0 × 10^–6^). Subjects were removed for excessive missingness (>0.02), for being identical or closely related to any subject in any sample (*p̂* > .2 based on common autosomal SNPs), or if there was evidence for diverging ancestry. Ancestry was estimated using multidimensional scaling applied to 8549 SNPs directly genotyped in all samples and in approximate linkage equilibrium. Imputation was performed using Beagle 3.0.440 (31) with the CEU HapMap3 data (32) to impute 1,235,109 autosomal SNP allele dosages. The first 20 ancestry-informative principal components were included as covariates, along with an indicator for each study.

### Genome-wide Association Analysis

Genome-wide association analysis was performed in PLINK using logistic regression to test the association between case-control phenotype and imputed SNP dosages under an additive model (33). Genotyping coordinates are given in NCBI Build 36/UCSC hg18 (National Center for Biotechnology Information, Bethesda, MD). Quality control was conducted separately for each sample. To test for SNPs associated with distinct aspects of MDD based on AAO, we performed three hypothesis-driven analyses (Supplemental Figure S2). The first analysis targeted those SNPs associated with early-onset MDD in a series of genome-wide association analyses, initially looking at the earliest onset cases (O1) against all control subjects, then the combined O1 and O2 cases against control subjects, then O1 to O3, etc., until all cases were included. This approach was based on the sequential additions method (34), which was developed to account for a quantitative trait that is measured in cases but not control subjects and provide an estimate of the best phenotype definition for future studies. The second analysis was similarly performed to examine those SNPs associated with later-onset MDD, but reversing the procedure (i.e., first looking at the latest-onset cases [O8] against all control subjects, then O7–O8 against control subjects, then O6–O8, etc., until all cases were included). The third analysis tested whether the extremes of AAO, both early and late, were introducing heterogeneity to the cases excluding O1 to O2 and O7 to O8, leaving those cases with onset within the interquartile range of AAO for each study (O3–O6). We then tested O3 to O6 cases against all control subjects. Each analysis was performed using all cases, male cases only, female cases only, and recurrent cases only. Analyses of all, or almost all, cases (O1–O8, O1–O7, or O2–O8) were used to identify SNPs that reached greatest significance without an AAO-specific effect and so were omitted from further analysis as would have been captured in the primary analysis of this data. In total, 52 genome-wide analyses were performed [= (6 + 6 + 1) × 4], making the standard multiple testing threshold of *p* < 5.0 × 10^–8^ for genome-wide significance anticonservative. We applied a Bonferroni correction for 52 analyses to genome-wide significance, which is highly conservative because many analyses were highly correlated. For replication, we selected SNPs with *p* < 9.5 × 10^–10^ in the discovery sample in more than one analysis (either by sex or recurrence or inclusion of octiles); the combination of cases that yielded the greatest significance was chosen as the basis for replication. This analysis strategy uses AAO as a stratifying variable to construct subsets of cases that may be more homogeneous and identify SNPs that are associated with susceptibility to MDD with a restricted AAO; it does not identify SNPs that control MDD AAO, which would require a case-only analysis of AAO as a quantitative trait.

### Replication Analysis

Five replication samples used in the primary analysis of this dataset had AAO information available [TwinGene (35), GenREDII/DepGenesNetwork (25), deCODE (9), PsychCoLaus (36), SHIP-LEGEND (Study of Health in Pomerania--Life-Events and Gene-Environment Interaction in Depression) (37)]. The GenPod/NEWMEDS and Harvard i2b2 studies, which appeared in the replication of the primary analysis of this dataset, did not have AAO data available and were not included. Four new replication studies were available: 1) a collection of samples available through the University of Mu¨nster (38, 39); 2) a combination of RADIANT cases from Denmark (40), the Danish DEMO and PRISME studies of MDD (41, 42), and a set of Danish population control subjects; 3) the CONVERGE (China Oxford and VCU Experimental Research on Genetic Epidemiology) study of MDD cases and control subjects recruited in China (18, 43, 44); and 4) the Generation Scotland study, which included measures on MDD (45). These are outlined, alongside the definitions of AAO, in Supplemental Methods. Due to the early median AAO in the GenREDII/ DepGenesNetwork, an artificial “median” was introduced at age 27 years, based on the median for the discovery samples. Those SNPs that passed our threshold of *p* < 9.5 × 10^–10^ in the discovery sample were tested for association within these nine replication studies. Due to differences in availability of genome-wide genotype data, each study was genotyped and/or imputed separately. A fixed-effect inverse variance based meta-analysis of the replication studies was performed using METAL (46).

### Polygenic Analysis

We also examined the association between early- and late-onset MDD and polygenic risk scores for other psychiatric disorders, as this might reflect either shared genetic etiology or phenotypic contamination (47). Polygenic risk scores for schizophrenia (9379 cases and 7736 control subjects) and bipolar disorder (6990 cases and 4820 control subjects) were created by the PGC, using imputed data and removing overlapping control subjects, ensuring that the datasets were completely independent (10, 11). Alzheimer’s disease polygenic risk scores were obtained from the GERAD (Genetic and Environmental Risk for Alzheimer’s disease) Consortium (48) and the coronary artery disease scores from the CARDIoGRAM Consortium (49). The imputed GERAD sample comprised 3177 cases and 7277 control subjects, and the CARDIoGRAM consortium consisted of 22,233 cases and 64,762 control subjects. All four disorders were chosen for their previous genetic and epidemiological evidence of overlap with MDD (50, 51, 52).

Polygenic risk scores were calculated for MDD cases and control subjects, summing the number of risk alleles carried, weighted by the natural log of their odds ratio in the original genome-wide association study (GWAS). Score SNPs with low minor allele frequency (<0.02) or in the major histocompatibility complex region were removed, and score datasets were pruned for linkage disequilibrium using the clumping command in PLINK to remove SNPs within 500 kb and *r*^2^ > .25 with a more significantly associated SNP. Seven scores were calculated, using a *p* value threshold (*P*_T_) to restrict to the most significant SNPs in their original genome-wide association analysis (*P*_T_ < .01, .05, .1, .2, .3, .4, .5). Logistic regression was used to test for association between polygenic risk scores and case-control status using four different case-control sets (all MDD cases vs. control subjects, O1–O3 cases vs. control subjects, O6–O8 cases vs. control subjects, and O1–O3 cases vs. O6–O8 cases) with 20 population principal components and study indicators as covariates. We calculated the proportion of variance explained (Nagelkerkeʼs *R*^2^) by subtraction of a full model (covariates + polygenic risk score) from a reduced model (covariates only). The GenRED sample was not included in the analysis as they had no cases within the O6–O8 analysis; the Bonn-Mannheim study was not included in the analysis of Alzheimer’s disease or coronary artery disease due to overlapping control subjects with the respective consortia.

## Results

### Summary of AAO

**Figure 1:**
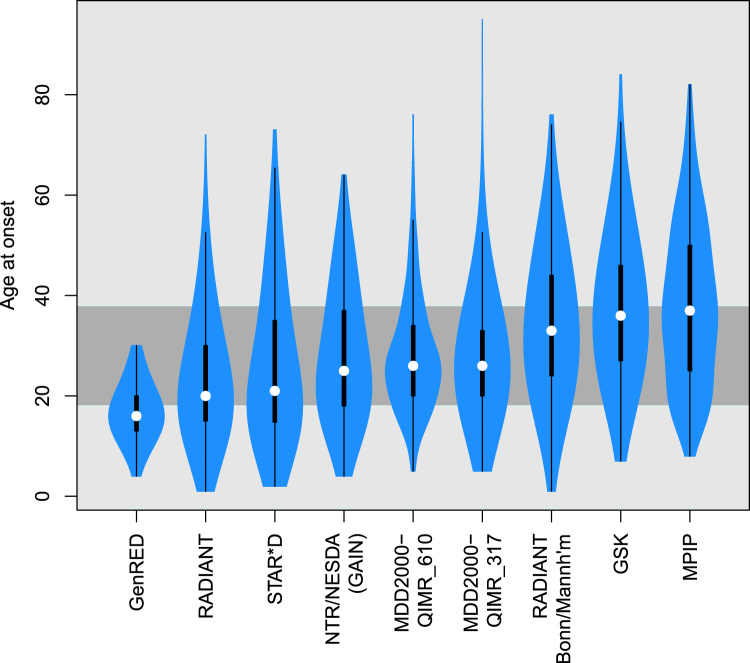
Distribution of age at onset across the nine studies included in the discovery analysis. Mid-gray band shows interquartile range across all studies excluding GenRED (Genetics of Recurrent Early-Onset Depression), which recruited only cases onset at 30 years or less. GAIN, Genetic Association Information Network; GSK, GlaxoSmithKline; MPIP, Max Planck Institute of Psychiatry; NESDA, Netherlands Study of Depression and Anxiety; NTR, Netherlands Twin Register.

After quality control, the sample consisted of 8920 MDD cases with AAO information and 9519 control subjects with 1,235,109 SNPs. The median AAO across all cases excluding GenRED was 27 years old (interquartile range: 18, 38) and the mean 28.9 ±13.64, reflecting the long tail of older onset cases (Figure 1 and Supplemental Figure S3). Median AAO by study ranged from 20 to 37 years (16 for GenRED, which recruited only recurrent cases with onset no greater than 30), with the German samples having a slightly older onset. Mean AAO was lower for recurrent than nonrecurrent cases, 27.1 to 33.0 (*p* < .001, correcting for study and excluding GenRED). AAO was also older for male subjects with a mean of 29.0 compared with 26.7 in female subjects (*p* < .001).

### Genome-wide Association Analysis

**Figure 2:**
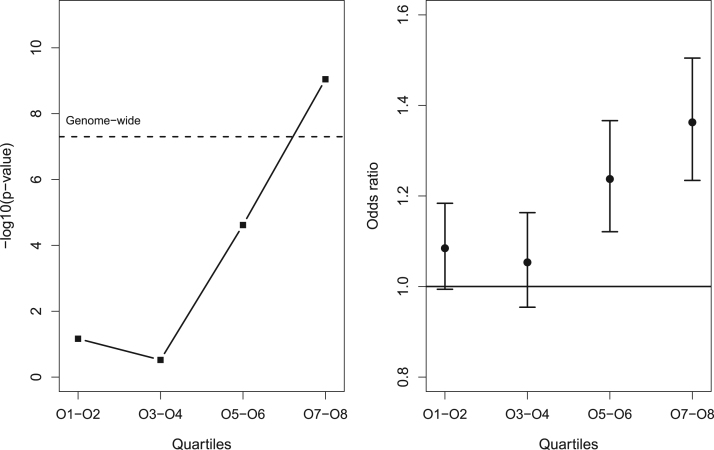
Evidence for association and effect size for rs7647854 on chromosome 3, with cases split into nonoverlapping quartiles by age at onset within discovery studies. O, octile.

Our analysis of both early- and late-onset octiles and the intermediate median of AAO excluding extreme-AAO cases led to four tests passing our significance cutoff for replication (*p* < 9.5 × 10^–10^), all for associations with SNP rs7647854 on chromosome 3. The only genome-wide significant association for this SNP was in the 50% oldest onset cases against all control subjects (O5–O8: *p* = 3.4 × 10^-11^) (Supplemental Figures S4 and S5). As a secondary analysis of this SNP, we split cases within our discovery sample into nonoverlapping quartiles and analyzed them against control subjects. This showed a strong association in the oldest quartile (O7–O8: *p* = 9.0 × 10^–10^, odds ratio [OR]: 1.37, 95% confidence interval [CI]: 1.23–1.51) (Figure 2), a moderate effect in the third quartile (O5–O6: *p* = 2.0 × 10^–5^, OR: 1.23), and no evidence of association in the two youngest quartiles (O1-O2: *p* = .07, OR: 1.09; O3-O4: *p* = .30, OR: 1.06). Furthermore, when the inclusion threshold for AAO was increased 1 year at a time within each study (until only 100 cases remained), we observed a gradual increase in effect size for rs7647854 in all studies as the analysis was restricted to progressively later onset cases (Supplemental Figure S6). Analysis by sex or recurrence revealed no additional findings.

### Replication Analysis

Given the results from our discovery dataset, rs7647854 was taken forward for replication in nine independent studies (Table 1). The SNP was tested for association with oldest half of MDD cases (O5–O8), because this analysis attained the smallest *p* value in the discovery studies. The SNP was either directly genotyped or imputed at high confidence across all studies (Supplemental Table S1). The SNP was significantly associated with MDD in a meta-analysis of these studies, with a *p* value of 7.5 × 10^–4^ and an OR of 1.10 (total number of MDD cases = 6107 and total number of control subjects 124,230) (Table 2; Supplemental Figure S5). Meta-analysis of the combined discovery sample with individual replication studies gave a *p* value of 5.2 × 10^–11^ and an OR of 1.16 (95% CI: 1.11–1.21), surpassing genome-wide significance.

### Polygenic Analysis of Comorbid Illnesses

**Figure 3:**
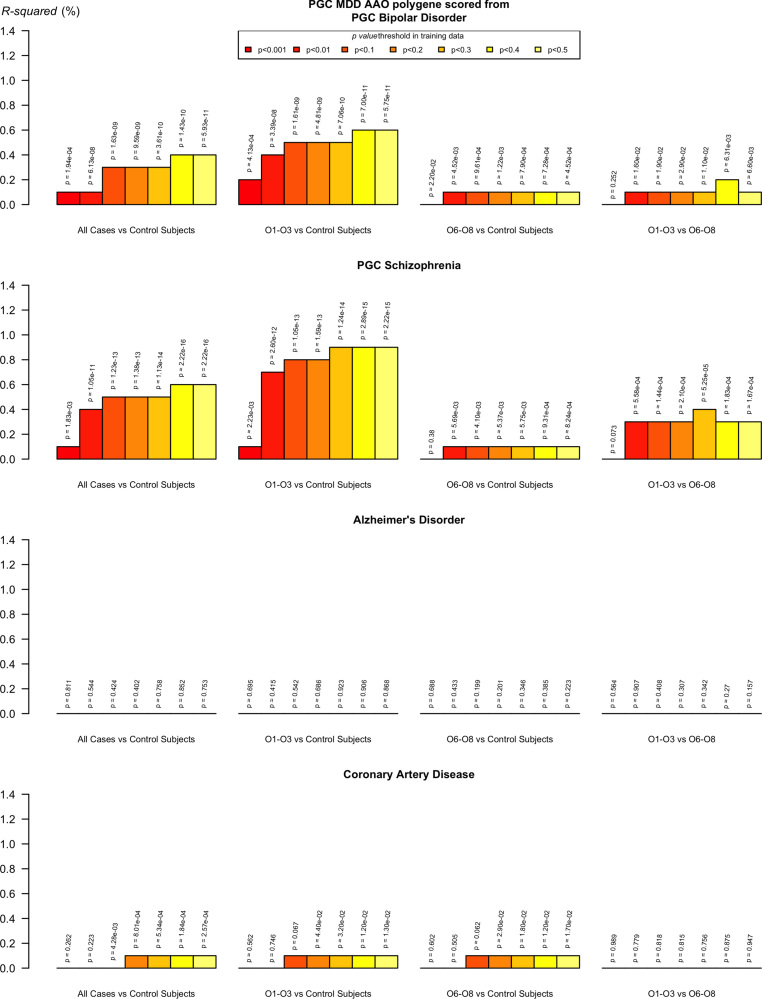
Polygenic risk profile scoring analysis of bipolar disorder, schizophrenia, Alzheimer’s disease, and coronary artery disease within the major depressive disorder (MDD) discovery studies (excluding GenRED [Genetics of Recurrent Early-Onset Depression]). We calculated the proportion of variance explained (Nagelkerkeʼs *R*^2^) by subtraction of a full model (covariates + polygenic risk score) from a reduced model (covariates only). AAO, age at onset; O, octile; PGC, Psychiatric Genomics Consortium.

In the PGC-MDD discovery studies, polygenic risk scores for BPD and SCZ were significantly associated with early-onset MDD. Restricting to only early-onset cases (O1-O3) versus control subjects, the amount of phenotypic variability explained (BPD: *R*^2^ = .41%, *p* = 1.4 × 10^–12^; SCZ: *R*^2^ = .67%, *p* = 3.0 × 10^–19^) was much greater than for later onset (O6–O8) cases (BPD: *R*^2^ = .16%, *p* = 1.9 × 10^–5^; SCZ: *R*^2^ = .14%, *p* = 3.9 × 10^–5^) (Figure 3). A similar increase in association with late-onset comorbid disorders was not seen. Polygenic risk scores for coronary artery disease from the CARDIoGRAM Consortium (49) were weakly positively associated with MDD, but this was consistent across early and late onset cases, in contrast to BPD and SCZ (coronary artery disease: O1–O3 cases vs. control subjects, *R*^2^ = .05%, *p* = .01; O6–O8 cases vs. control subjects, *R*^2^ = .05%, *p* = .01; O1–O3 cases vs. O6–O8 cases, *R*^2^ ≤ .01%, *p* = .76) (Figure 3). No association with MDD status was seen for the scores generated for Alzheimer’s disease from the GERAD Consortium (48) regardless of AAO (O1–O3 cases vs. control subjects, *R*^2^ < .01%, *p* = .868; O6–O8 cases vs. control subjects, *R*^2^ = .02%, *p* = .223; O1–O3 cases vs. O6–O8 cases, *R*^2^ = .03%, *p* = .157) (Figure 3). Full results from these analyses are available in Supplemental Tables S2 and S3.

## Discussion

Our analysis of AAO and the genetic architecture of MDD suggested that AAO-specific genetic risk factors exist. Unexpectedly, the strongest associations we observed were with the oldest half of MDD cases, where previous analyses have focused on early-onset MDD, supported by studies of the genetic epidemiology of MDD. We emphasize that here “late”-onset MDD was at still a relatively young age (median onset of MDD was at age 27 years with 98% of our sample having onset before 60 years) rather than onset in old age. It is clearer to conceptualize later-onset cases here as “adult-onset” cases, and we recommend that this subgroup should be included in recruitment of future genetic studies, especially as no difference in the heritability captured by SNPs was observed for this subtype compared with early-onset cases (see Supplemental Table S3). However, substantial differences in AAO across studies make it difficult to put a precise age cutoff for this recommended adult-onset MDD.

We show significant association with rs7647854 on chromosome 3, which was associated with the 50% latest-onset cases (OR: 1.16, *p* = 5.2 x 10^–11^). This SNP was identified as the second strongest association in the primary mega-analysis of the PGC-MDD dataset (9), though at a much lower significance, and did not replicate (discovery *p* = 6.5 × 10^–7^, replication *p* = .67). Stratifying by AAO leads to much stronger associations in both the discovery and replication samples, despite the reduced sample size. Although analyzing across multiple overlapping AAO subtypes runs the risk of overfitting to maximize significance, we observed highly significant associations for this SNP in both discovery and replication samples. Furthermore, we also observed a gradual increase in effect size as both raw AAO within studies and AAO percentile across studies increased (Figure 2, Supplemental Figure S4). That this was seen both for raw AAO within studies and as a percentile, and in both European and Chinese ancestry studies across a wide variety of AAO measurement tools, suggests that whereas our cutoff of the 50% latest onset cases is arbitrary, the effect exists regardless of exact cutoff used. However, this does not rule out the possibility that AAO may be a proxy for a more homogenous subgroup of MDD based on another factor (e.g., an age-specific environmental trigger or distinct pattern of symptoms with onset later in life). That median AAO and effect size across studies was not significantly correlated (*r* = .011, *p* =.68) despite greater effect sizes by AAO percentile within studies suggests that differences in measurement of AAO across studies might obscure effects. rs7647854 is intergenic, with flanking genes including *C3orf70*, *VPS8*, *EHHADH,* and *MAP3K13. C3orf70*, *VPS8*, and *MAP3K13* all show evidence of expression in the brain in several areas of potential interest for MDD including the hypothalamus, frontal cortex, pituitary, and thyroid (GTEx; http://www.gtexportal.org/) (53). *VPS8* and *MAP3K13* also show a slight increase in RNA expression within various brain regions during neonatal development (Human Brain Transcriptome; http://hbatlas.org/) (54) with maintained expression into adulthood.

This genetic association arises with the supposedly less heritable form of adult-onset MDD, although such summary measures give no information on the effect sizes of individual SNPs. One potential explanation is greater contamination of early-onset MDD cases by individuals misclassified with MDD or having comorbid disorders. Longitudinal studies show that early-onset depressive symptoms predict not only adult depression but also psychosis [e.g., (55)], and there is significant genetic overlap between MDD and other psychiatric disorders (50). Inclusion of early-onset cases with individuals who will later develop SCZ or BPD would reduce the power of GWASs for MDD, though secondary analysis of age at interview did not support this (Supplemental Figure S7). The polygenic risk score results show that early- and adult-onset MDD cases differ in their genetic susceptibility to BPD and SCZ and suggest that some of the heterogeneity in MDD results from the inclusion of early-onset cases with a greater genetic overlap or misclassification with these disorders. We did not observe a similar pattern of association for two disorders that are often comorbid with late-onset MDD, Alzheimer’s disease, and coronary artery disease (51, 52). We did, however, show for the first time a genetic overlap between MDD and coronary artery disease irrespective of AAO but no overlap between MDD and Alzheimer’s disease. We also found that the heritability explained by SNPs across the genome for early- and late-onset MDD did not significantly differ, suggesting, for common variants at least, that recruiting only early-onset cases would not increase power (Supplemental Table S3).

The limitations of our approach are the reduced sample size and multiple testing from stratifying cases into subtypes, and requiring potentially less reliable secondary phenotypes to be widely collected. Measures such as AAO rely on self-report and are often assessed differently across studies, which can be problematic for comparison. The analyses presented here addressed this by analyzing AAO relative to the median of a study, though this assumed that each study recruited cases from the same distribution of onset with observed differences due to how AAO was defined. Furthermore, the effect of our genome-wide associated SNP were consistent in the replication studies, ethnicities, and countries, suggesting that differences in measurement may not be as much of a limitation as expected, at least in the case of AAO in MDD. The other disadvantage of looking at more homogenous groups is the reduction in sample size. However, it has previously been shown that only modest increases in effect size may be required to offset the reduction in power from analyzing fewer cases, implying that analyses of more homogenous subgroups have the potential to identify novel associations (56).

Our study illustrates the value of using additional phenotypic information on cases in GWASs. We show here that including information on AAO increases the power to detect associations with MDD, and that analyzing polygenic risk scores from related diseases enables us to identify sources of phenotypic heterogeneity that may have hampered previous genetic studies. In contrast to other approaches that weight cases on AAO (57), our stratification of cases on AAO is agnostic to the direction of the phenotypic effect. Our approach uses the additional phenotype data previously collected by genetic studies and complements an alternative emphasis on collecting large sample sizes through minimal phenotyping. Both strategies will undoubtedly be necessary to identify and characterize different components of the genetic architecture of psychiatric disorders. Furthermore, our analysis shows that tackling phenotypic heterogeneity in MDD can successfully lead to the discovery of a genetic risk factor despite reduced sample size. The identification here of a novel genetic risk variant for MDD is of great importance due to both the scarcity of evidence for the underlying biology and its pressing economic health burden.

## Figures and Tables

**Table 1:** Summary of MDD Discovery and Replication Cohorts

	Study	Country	Measure of AAO	Cases With AAO	Control Subjects	Median AAO
Discovery	NESDA/NTR (GAIN)	The Netherlands	CIDI	1675	1765	25
	GenRED	United States	DIGS3	1020	1253	16
	GSK	Germany	SCAN	887	864	36
	MDD2000-QIMR_610	Australia	CIDI/SSAGA	432	751	26
	MDD2000-QIMR_317	Australia	CIDI/SSAGA	1015	960	26
	MPIP	Germany	Asked at interview	373	537	37
	RADIANT Bonn/Mannheim	Germany	SCAN	883	1290	33
	RADIANT	United Kingdom	SCAN	1407	1588	20
	STAR*D	United States	Asked at interview	1228	511	21
	Total			8920	9519	
Replication	TwinGene	Sweden	SALT	1009	8601	40
	PsyCoLaus	Switzerland	DIGS	1358	1687	33
	SHIP-LEGEND	Germany	M-CIDI	381	1827	37
	GenRED2/DepGenesNetworks	United States	DIGS3	1296	930	17
	University of Mu¨nster	Germany	SCID	402	516	27
	Combined Danish sample	Denmark	SCAN	461	1197	31
	CONVERGE	China		5715	5537	34
	deCODE	Iceland	Hospital records	1005	99,175	39
	Generation Scotland	United Kingdom	SCID	1611	4760	30
	Total			13,238	124,230	
Total				22,158	133,749	

AAO, age at onset; CONVERGE, China Oxford and VCU Experimental Research on Genetic Epidemiology; CIDI, Composite International Diagnostic Interview; DIGS, Diagnostic Interview for Genetic Studies; GAIN, Genetic Association Information Network; GenRED, Genetics of Recurrent Early-Onset Depression; GSK, GlaxoSmithKline; M-CIDI, Munich-Composite International Diagnostic Interview; MDD, major depressive disorder; MPIP, Max Planck Institute of Psychiatry; NESDA, Netherlands Study of Depression and Anxiety; NTR, Netherlands Twin Register; SALT, Screening Across the Lifespan Twin; SCAN, Schedules for Clinical Assessment in Neuropsychiatry; SCID, Structured Clinical Interview for DSM Disorders; SHIP-LEGEND, Study of Health in Pomerania--Life-Events and Gene-Environment Interaction in Depression; SSAGA, Semi-Structured Assessment for the Genetics of Alcoholism; STAR*D, Sequenced Treatment Alternatives to Relieve Depression.

**Table 2:** Summary of Association With rs7647854, Located at 3q27.2 (186359477 Base Pairs), With Odds Ratio for the Reference Allele, G (Frequency 0.16) Compared With the Nonreference Allele A

Study	MDD Cases, *n*	Control Subjects, *n*	OR (95% CI)	*p* Value
Discovery	3869	9519	1.30 (1.20–1.40)	3.4 × 10^–11^
Replication	6107	124,230	1.10 (1.05–1.17)	7.5 × 10^–4^
Meta-analysis	9976	133,749	1.16 (1.11–1.21)	5.2 × 10^–11^

All results reported are for the oldest half of MDD cases (O5-8), which had the strongest evidence for association in the discovery study.

CI, confidence interval; MDD, major depressive disorder; OR, odds ratio.
