# Supplementary material for: Genome-wide Association for Major Depression Through Age at Onset Stratification: Major Depressive Disorder Working Group of the Psychiatric Genomics Consortium
Source: Biol Psychiatry. 2017 Feb 15;81(4):325–35. doi: 10.1016/j.biopsych.2016.05.010 (PMC5262436; doi:10.1016/j.biopsych.2016.05.010)
Supplement: Supplementary file 1 — Supplementary material [file mmc1.pdf]

Genome-wide Association for Major Depression Through Age at Onset Stratification

Supplemental Information

Contents

Supplemental Figures 2
Figure S1. Cumulative distribution of AAO in discovery studies 2
Figure S2. Analysis strategy of MDD cases by overlapping subsets of AAO. 3
Figure S3. Distribution of age at onset in discovery samples 4
Figure S4. Manhattan, QQ, and SNAP plots for rs76478545 5
Figure S5. Forest plot of effect sizes (odds ratios) for rs7647854 6
Figure S6. By-study association analysis of rs7647854 7
Figure S7. Polygenic risk score of schizophrenia and bipolar disorder within MDD stratifying by both age at onset and age at interview 8
Supplemental Tables 9
Table S1. Genotyping and Imputation status of rs7647854 by study with info score. 9
Table S2. Results from polygenic score analysis 10
Table S3. Results of the GCTA analysis 11
Table S4. Associations of rs7647854 with other conditions. 11
Supplemental Methods 11
Genome-wide complex trait analysis 11
Description of replication studies 12
PsyCoLaus 12
SHIP-LEGEND 12
TwinGene 13
Genetics of Recurrent Early-Onset Depression Phase II & Depression Genes and Networks 13
Danish replication sample 14
University of Münster Depression Cohorts 16
deCODE 17
CONVERGE 17
Generation Scotland 17
GERAD1 Consortium study description 18

GERAD1 Consortium authorship 19
CONVERGE Consortium authorship 20
CARDIoGRAM Consortium authorship 23
Supplemental References 28

Supplemental Figures

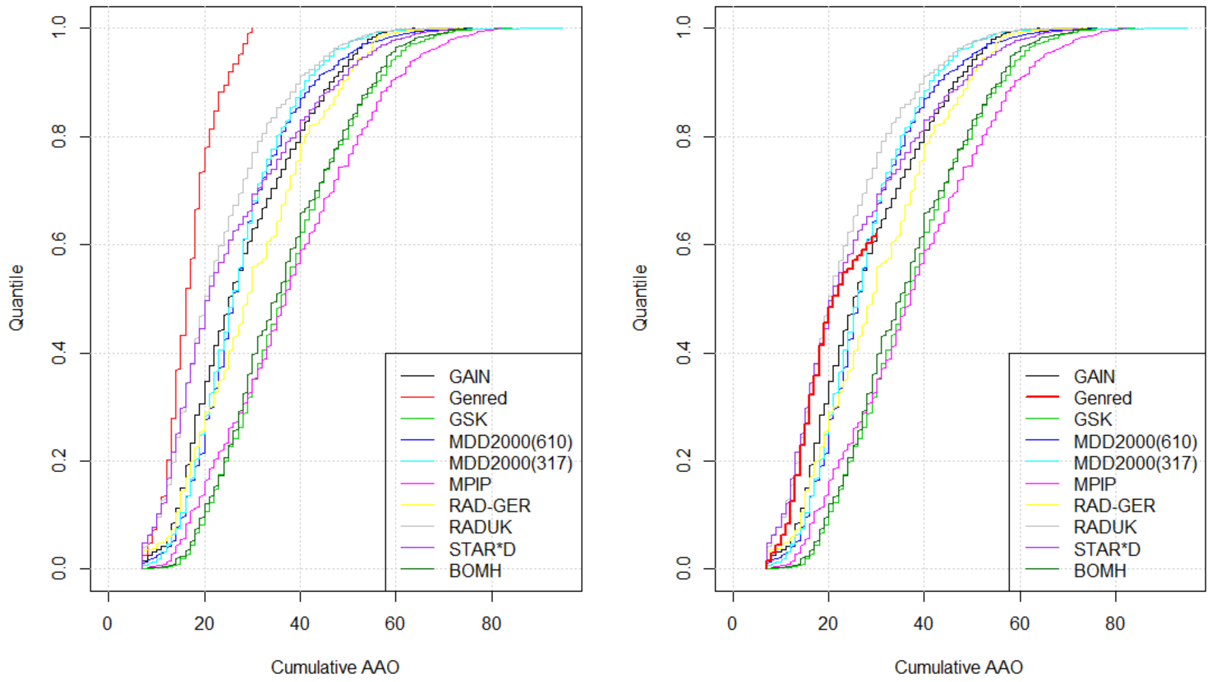

Figure S1. Cumulative distribution of AAO in discovery studies, showing how GenRED distribution (red line) was scaled due to ascertainment on age at onset of 30 or less.

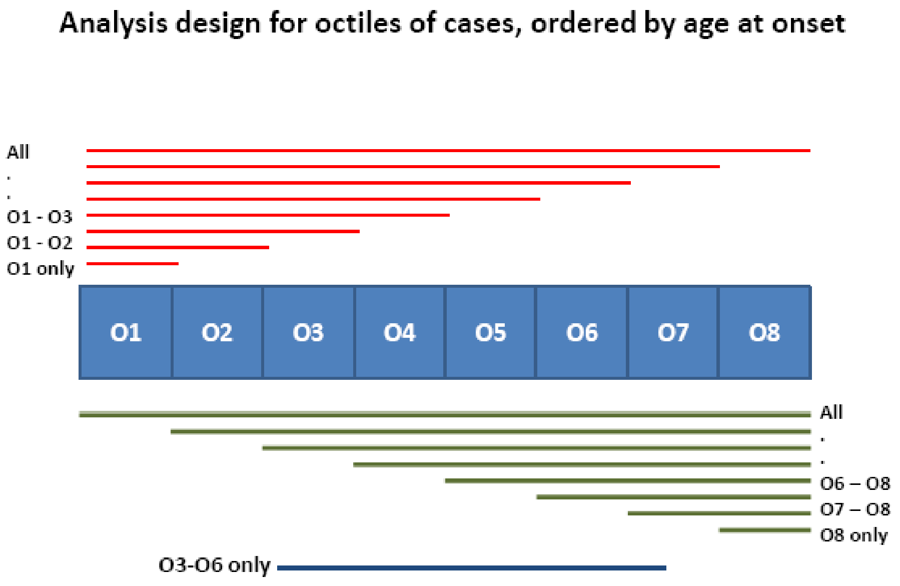

Figure S2. Analysis strategy of MDD cases by overlapping subsets of AAO.

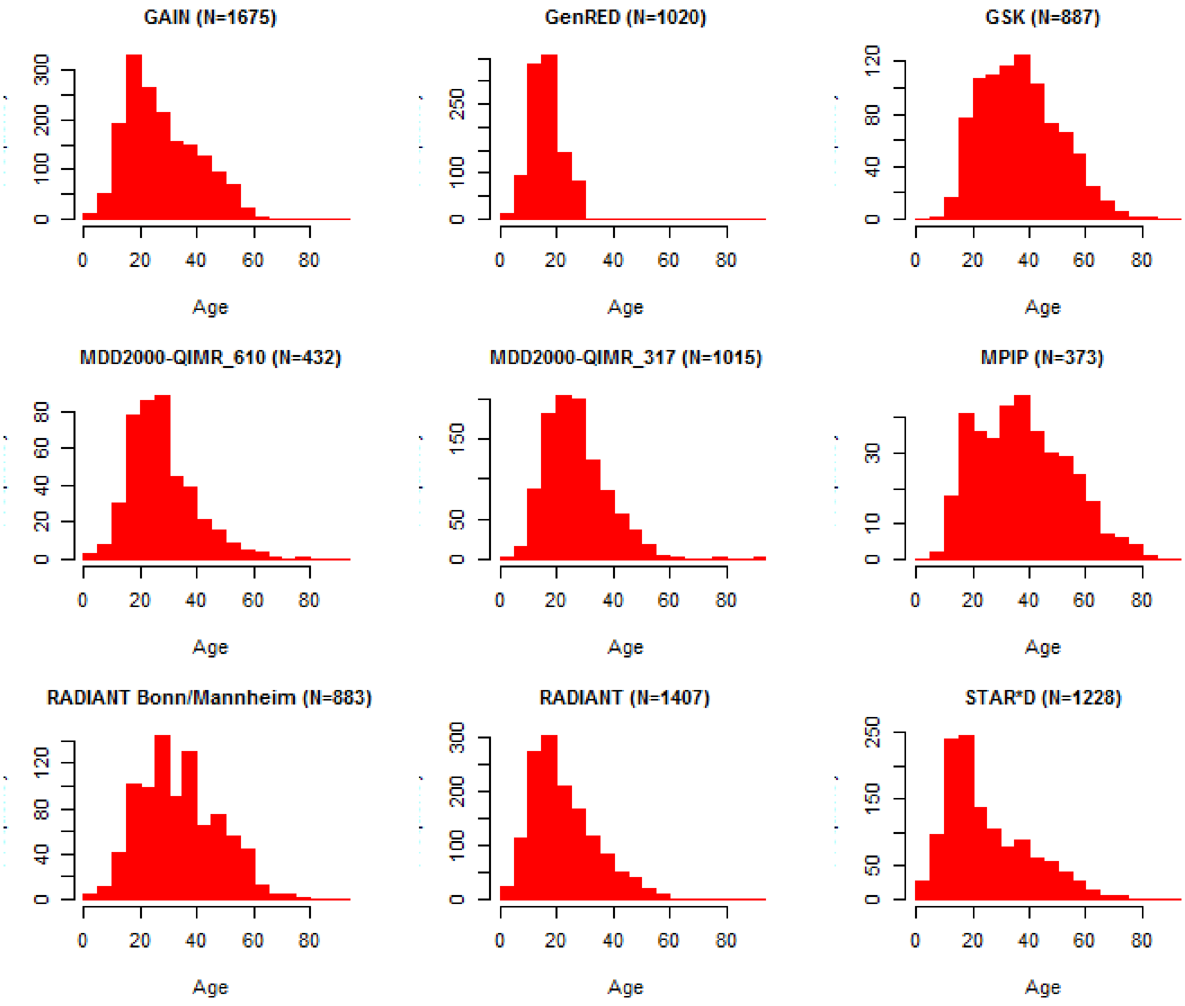

Figure S3. Distribution of age at onset in discovery samples.

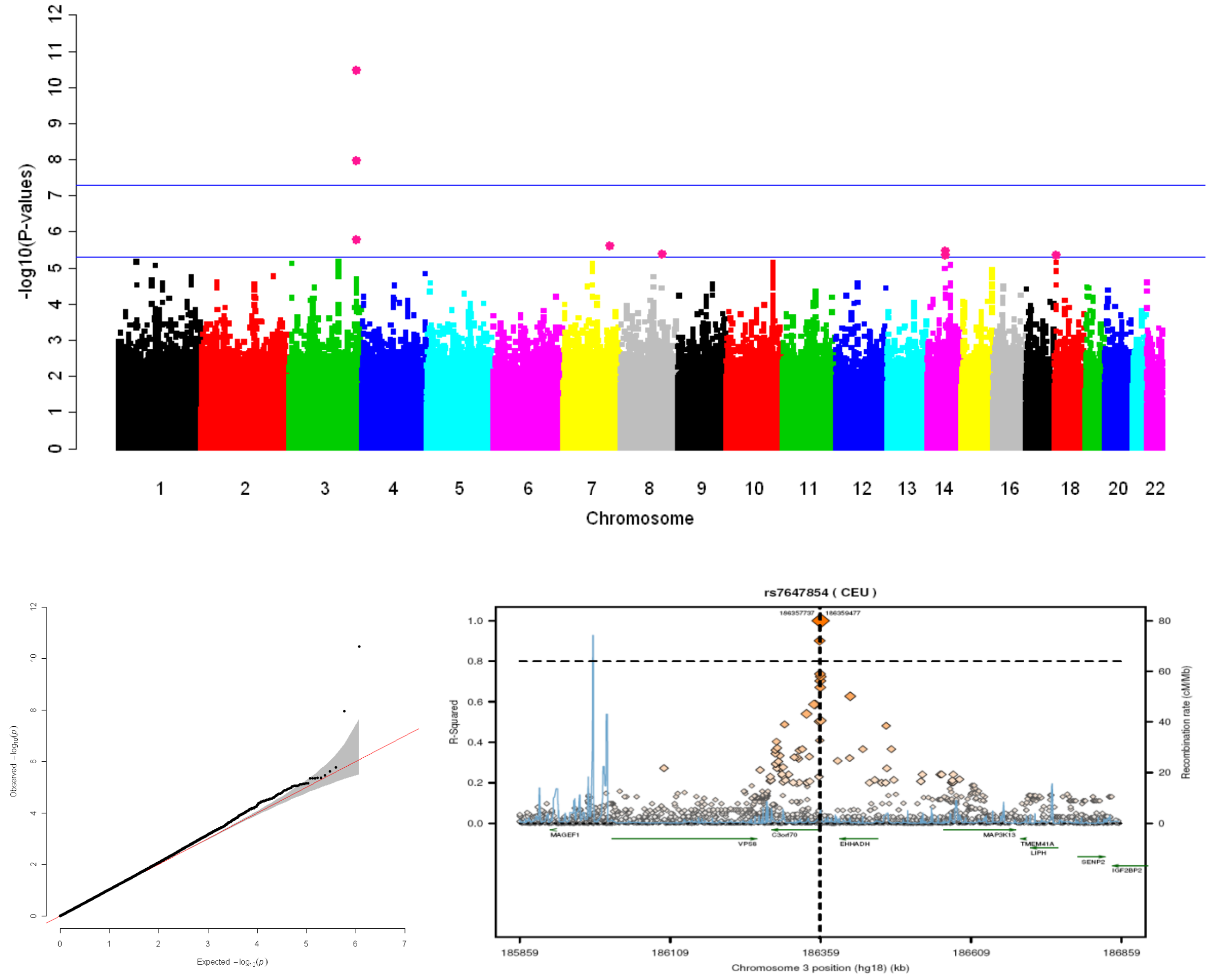

Figure S4. Manhattan, QQ, and SNAP plots for rs7647854 in O5-8 of the discovery samples.

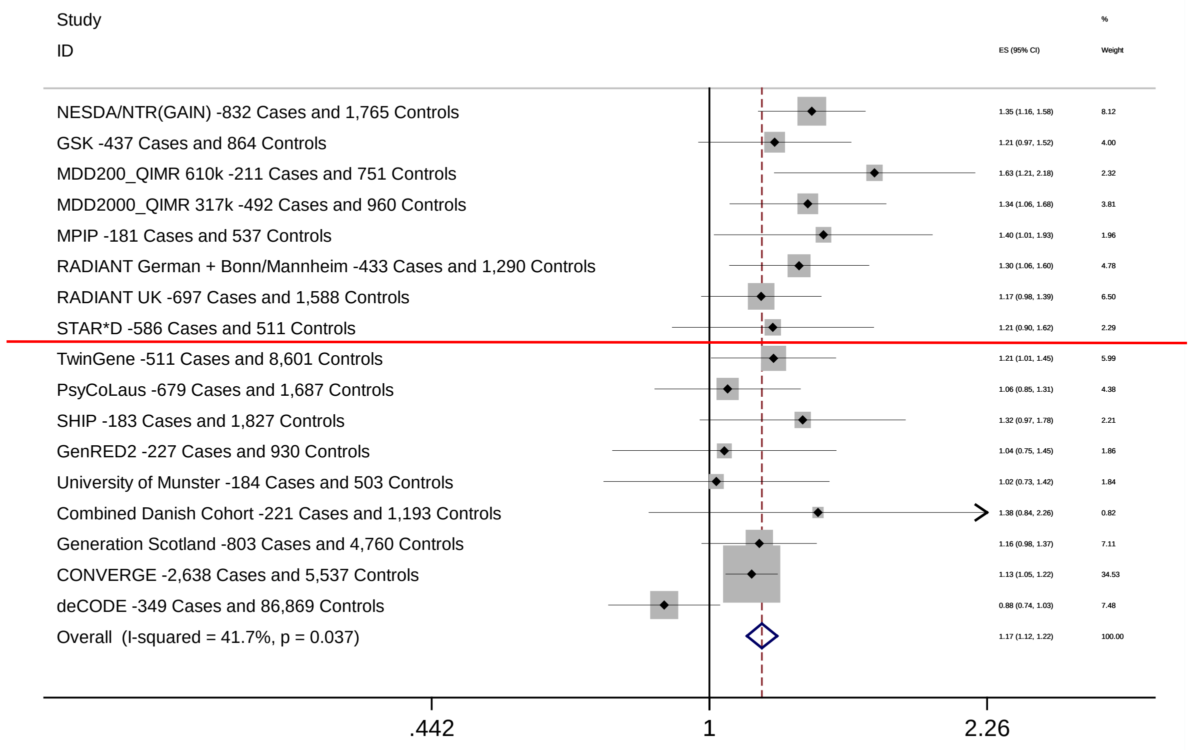

Figure S5. Forest plot of effect sizes (odds ratios) for rs7647854 in 50% latest-onset cases against controls across eight discovery studies (above horizontal line; GenRED not included) and nine replication studies (below line).

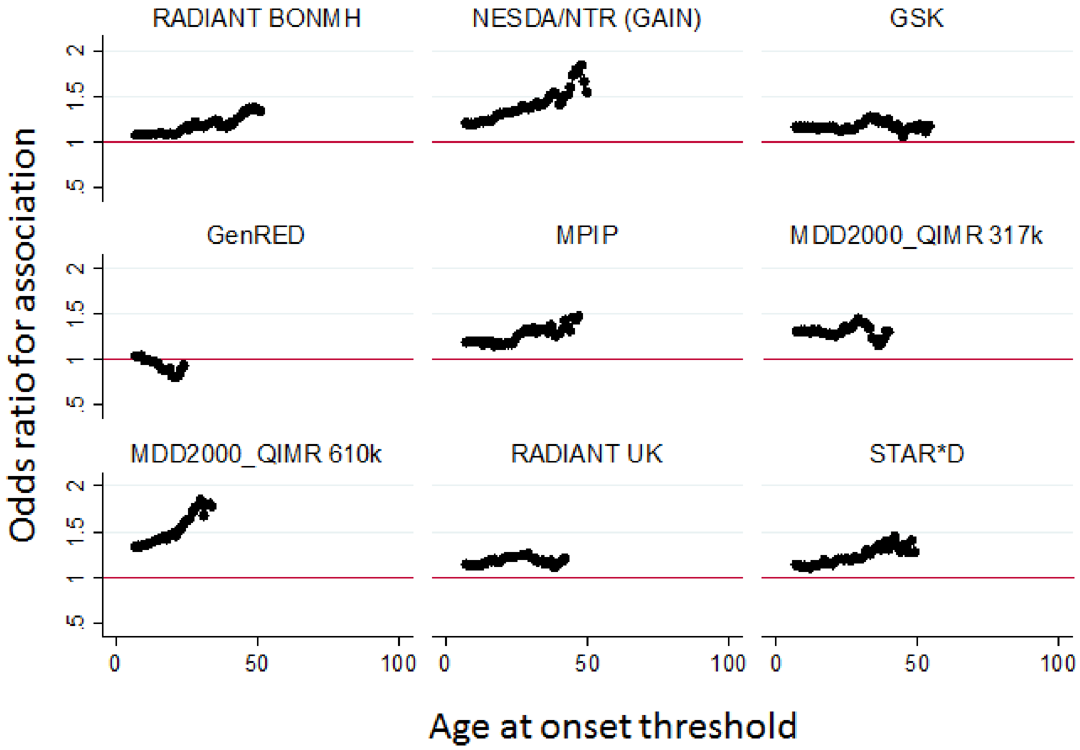

Figure S6. By-study association analysis of rs7647854, following the one-year sequential addition method of Macgregor *et al.* (1)**.** For each discovery study, the figure shows the odds ratio for association in a case control study, where only cases onset above the AAO indicated on the x-axis are included in the analysis. Analysis was terminated when fewer than 100 cases remained. Analysis shows consistency of effect across studies, with the OR increasing when only older-onset cases are included.

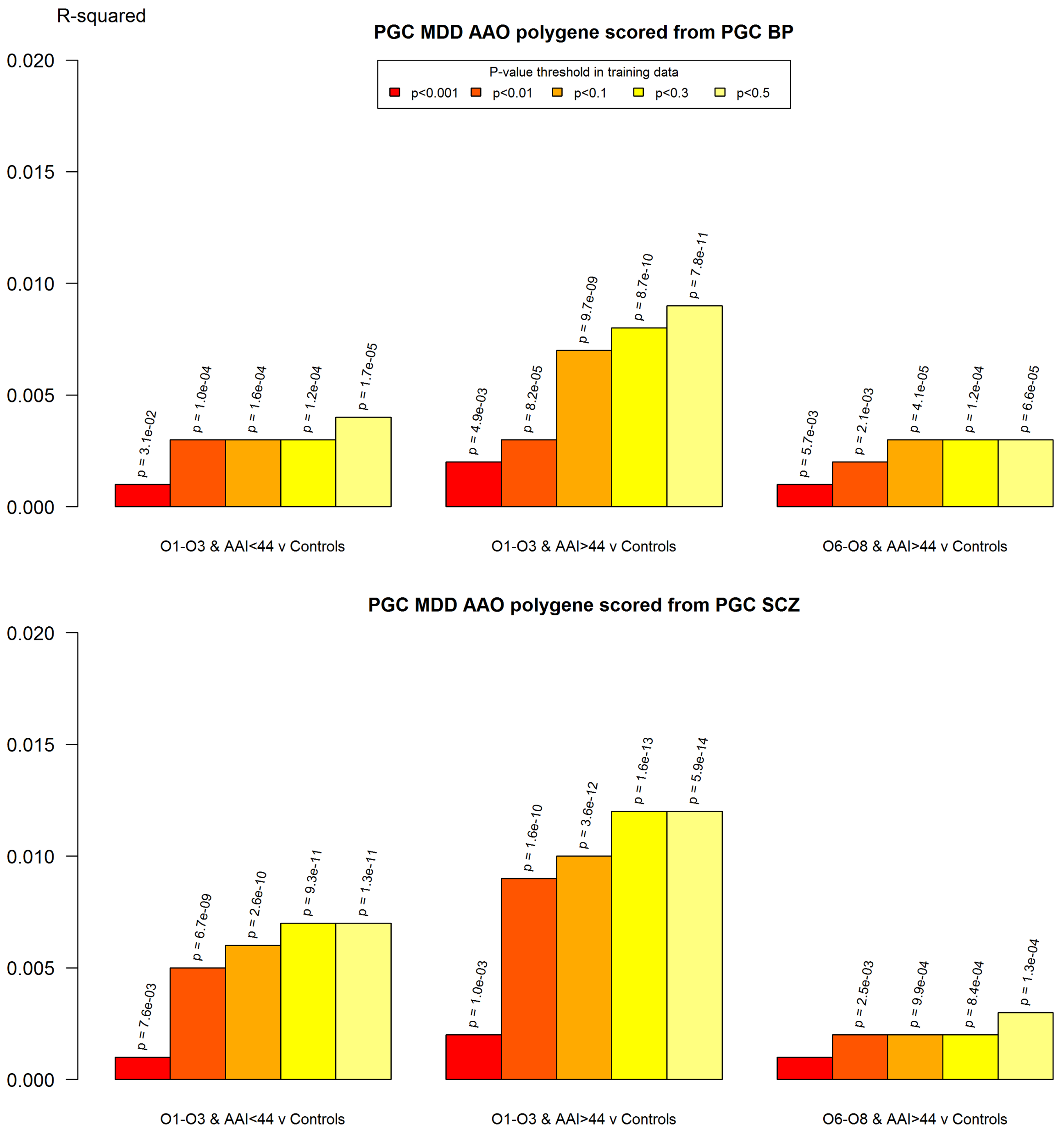

Figure S7. Polygenic risk score of schizophrenia and bipolar disorder within MDD stratifying by both age at onset and age at interview (AAI). The results show the increased overlap with bipolar disorder (BP) and schizophrenia (SCZ) in early-onset MDD (O1-3) was not driven by cases with earlier AAI (in fact the opposite). This tentatively suggests it reflects true genetic overlap rather than misdiagnosed MDD cases that eventually develop SCZ or BP. AAI of 44 was used a cut-off as this was the median of the sample. We calculated the proportion of variance explained (Nagelkerke's R^2) by subtraction of a full model (covariates + RPS) score from a reduced model (covariates only).

Supplemental Tables

Table S1. Genotyping and Imputation status of rs7647854 by study with info score.

Stage	Study	Genotyped?	Info
Discovery	GAIN	Genotyped	N/A
Discovery	GSK	Genotyped	N/A
Discovery	MDD2K_600k	Mixed	0.89
Discovery	MDD2K_317k	Mixed	0.81
Discovery	MPIP	Genotyped	N/A
Discovery	RADIANT_Bonn/Mannheim	Genotyped	N/A
Discovery	RADIANT_UK	Genotyped	N/A
Discovery	STAR*D	Imputed	0.62
Replication	TwinGene	Genotyped	N/A
Replication	PsyCoLaus	Imputed	0.8959
Replication	SHIP-LEGEND	Imputed	0.87
Replication	GenRED2/DepGenesNetworks	Genotyped	0.94
Replication	deCODE	Mixed	0.997
Replication	University of Münster	Genotyped	N/A
Replication	Combined Danish	Mixed	1.01
Replication	Generation Scotland	Genotyped	N/A
Replication	Converge	Mixed	0.98

Table S2. Results from polygenic score analysis using the genetic association results from the PGC Bipolar Disorder (2), PGC Schizophrenia (3), GERAD1 Alzheimer's disease (4), and CARDIoGRAM coronary heart disease (CAD) (5). GWAS to score into MDD discovery studies (excluding GenRED and Bonn-Mannheim). We calculated the proportion of variance explained (Nagelkerke's R^2 for logistic regression and R^2 for linear regression; reported as a percentage) by subtraction of a full model (covariates + RPS) score from a reduced model (covariates only). All results presented were corrected for sex, 20 population principal components and study indicators.

Subset	Bin	*p*-value Threshold	Bipolar Disorder		Schizophrenia		Alzheimer's Disease		CAD	
			R^2 (%)	*p*-value	R^2 (%)	*p*-value	R^2 (%)	*p*-value	R^2 (%)	*p*-value
Youngest 3/8 v. controls	1	0.001	0.2	$4.13*10^{-4}$	0.1	$2.23*10^{-3}$	<0.01	0.695	<0.01	0.562
	2	0.01	0.4	$3.39*10^{-8}$	0.7	$2.60*10^{-12}$	<0.01	0.415	<0.01	0.746
	3	0.1	0.5	$1.61*10^{-9}$	0.8	$1.05*10^{-13}$	<0.01	0.542	0.1	0.067
	4	0.2	0.5	$4.81*10^{-9}$	0.8	$1.59*10^{-13}$	<0.01	0.686	0.1	0.044
	5	0.3	0.5	$7.06*10^{-10}$	0.9	$1.24*10^{-14}$	<0.01	0.923	0.1	0.032
	6	0.4	0.6	$7.00*10^{-11}$	0.9	$2.89*10^{-15}$	<0.01	0.906	0.1	0.012
	7	0.5	0.6	$5.75*10^{-11}$	0.9	$2.22*10^{-15}$	<0.01	0.868	0.1	0.013
Oldest 3/8 v. controls	1	0.001	<0.01	0.022	<0.01	0.380	<0.01	0.688	<0.01	0.602
	2	0.01	0.1	$4.52*10^{-3}$	0.10	$5.69*10^{-3}$	<0.01	0.433	<0.01	0.505
	3	0.1	0.1	$9.61*10^{-4}$	0.10	$4.10*10^{-3}$	<0.01	0.199	0.1	0.062
	4	0.2	0.1	$1.22*10^{-3}$	0.10	$5.37*10^{-3}$	<0.01	0.201	0.1	0.029
	5	0.3	0.1	$7.90*10^{-4}$	0.10	$5.75*10^{-3}$	<0.01	0.346	0.1	0.018
	6	0.4	0.1	$7.28*10^{-4}$	0.10	$9.31*10^{-4}$	<0.01	0.385	0.1	0.012
	7	0.5	0.1	$4.52*10^{-4}$	0.10	$8.24*10^{-4}$	<0.01	0.223	0.1	0.017
All cases v. controls Cases only included if had age of onset information	1	0.001	0.1	$1.94*10^{-4}$	0.10	$1.83*10^{-3}$	<0.01	0.811	<0.01	0.262
	2	0.01	0.1	$6.13*10^{-8}$	0.40	$1.05*10^{-11}$	<0.01	0.544	<0.01	0.223
	3	0.1	0.3	$1.63*10^{-9}$	0.50	$1.23*10^{-13}$	<0.01	0.424	<0.01	$4.28*10^{-3}$
	4	0.2	0.3	$9.59*10^{-9}$	0.50	$1.38*10^{-13}$	<0.01	0.402	0.1	$8.01*10^{-4}$
	5	0.3	0.3	$3.61*10^{-10}$	0.50	$1.13*10^{-14}$	<0.01	0.758	0.1	$5.34*10^{-4}$
	6	0.4	0.4	$1.43*10^{-10}$	0.60	$2.22*10^{-16}$	<0.01	0.852	0.1	$1.84*10^{-4}$
	7	0.5	0.4	$5.93*10^{-11}$	0.60	$2.22*10^{-16}$	<0.01	0.753	0.1	$2.57*10^{-4}$
Youngest 3/8 v. Oldest 3/8	1	0.001	<0.010	0.252	<0.00	0.073	<0.01	0.564	<0.01	0.989
	2	0.01	0.1	0.016	0.30	$5.58*10^{-4}$	<0.01	0.907	<0.01	0.779
	3	0.1	0.1	0.019	0.30	$1.44*10^{-4}$	<0.01	0.408	<0.01	0.818
	4	0.2	0.1	0.029	0.30	$2.10*10^{-4}$	<0.01	0.307	<0.01	0.815
	5	0.3	0.1	0.011	0.40	$5.25*10^{-5}$	<0.01	0.342	<0.01	0.756
	6	0.4	0.2	$6.31*10^{-3}$	0.30	$1.83*10^{-4}$	<0.01	0.270	<0.01	0.875
	7	0.5	0.1	$6.60*10^{-3}$	0.30	$1.67*10^{-4}$	<0.01	0.157	<0.01	0.947
AAO as continuous trait	1	0.001	0.03	0.144	0.01	0.440	0.01	0.455	<0.01	0.692
	2	0.01	0.09	$8.16*10^{-3}$	0.09	0.010	<0.01	0.799	<0.01	0.781
	3	0.1	0.09	$7.82*10^{-3}$	0.11	$3.89*10^{-3}$	0.01	0.346	<0.01	0.595
	4	0.2	0.10	$6.07*10^{-3}$	0.10	$6.21*10^{-3}$	0.02	0.269	<0.01	0.636
	5	0.3	0.10	$4.55*10^{-3}$	0.13	$1.19*10^{-3}$	0.01	0.347	<0.01	0.765
	6	0.4	0.11	$2.83*10^{-3}$	0.11	$2.82*10^{-3}$	0.02	0.244	<0.01	0.852
	7	0.5	0.11	$3.21*10^{-3}$	0.12	$2.52*10^{-3}$	0.03	0.125	<0.01	0.954

Table S3. Results of the GCTA analysis estimating the variance captured by genotyped SNPs in early-onset cases (O1-3) and late-onset cases (O6-8). No difference in SNP-heritability was observed across early and late-onset MDD. A bivariate model looking at the overlap of heritability between early and late onset cases when both compared to controls separately gave a genetic correlation (r_G) of 99.77%.

		All Cases			Recurrent Cases		
**Cases**	**Controls**	**Cases**	**h_p^2 (SE)**	***p*-value**	**Cases**	**h_p^2 (SE)**	***p*-value**
O1-O3	9519	3969	0.220 (0.036)	$4x10^{-11}$	3120	0.245 (0.042)	$1x10^{-9}$
O6-O8	9519	2779	0.231 (0.046)	$1x10^{-7}$	1968	0.262 (0.059)	$2x10^{-6}$

Table S4. Associations of rs7647854 with other conditions.

SNP	Chr.	Ref. allele	Discovery		Alzheimer's GWAS		CAD GWAS	
			**OR**	***P***	**OR**	***P***	**OR**	***P***
rs7647854	3	G	1.30	$3.4x10^{-11}$	0.95	0.19	1.04	0.04

Supplemental Methods

Genome-wide Complex Trait Analysis

In order to estimate the heritability of AAO within MDD, Genome-wide Complex Trait Analysis (GCTA) was used to estimate the proportion of AAO phenotypic variance explained by common SNPs across the genome (6). SNPs were included with MAF > 0.01 and imputation R^2 > 0.6 in all nine studies, leaving a total of 962,903 SNPs. One individual was removed from each pair of individuals sharing more than 5% of their genetic material. Two analyses were run, analyzing cases in O1-3 against all controls and O6-8 cases against all controls. Each analysis was also run restricting to cases with recurrent depression only. Study indicators, twenty principal components and sex were included as covariates. Prevalence for MDD was set at 16.5% from Kessler *et al.* (8). A bivariate model was implemented within GCTA to examine the genetic covariance between early onset (O1-3) cases and later onset cases (O6-8). Controls were randomly split between the two groups and did not different significantly based on gender or originating study. Bivariate analysis was run covarying for 20 population principal components, study indicators and gender. Results are outlined in Table S1.

Description of Replication Studies

PsyCoLaus

Subjects were selected from subjects of European ancestry from a community survey (CoLaus) carried out in the city of Lausanne, Switzerland (9). Subjects were randomly selected from a complete list of the Lausanne inhabitants aged 35-75 years. All 35 to 66-year old participants were invited by letters also to participate in the psychiatric evaluation (PsyCoLaus) (10). Sixty-seven percent of the participants of the CoLaus study in the age range between 35-66 years accepted the psychiatric evaluation, which resulted in a sample of 3,719 individuals, of whom 92% were of European ancestry. Psychiatric assessment in the PsyCoLaus sub-study included the semi structured Diagnostic Interview for Genetic Studies (DIGS), French version (11). Cases met DSM-IV criteria for MDD and controls were devoid of any psychiatric disorders. A subset of the 3,419 European subjects who received full psychiatric assessment and gave consent for genetic testing were selected for GWAS genotyping. This research was approved by the local institutional review board. All participants received a detailed description of the goal and funding of the study and signed a written informed consent.

SHIP-LEGEND

Data from the Study of Health in Pomerania (SHIP) were used (12; 13). The target population was comprised of adult German residents in northeastern Germany living in 3 cities and 29 communities, with a total population of 212,157. A two-stage stratified cluster sample of adults aged 20-79 years (baseline) was randomly drawn from local population registries. The net sample (without migrated or deceased persons) comprised 6,267 eligible subjects of whom 4,308 Caucasian subjects participated at baseline SHIP-0 between 1997 and 2001. Follow-up examination (SHIP-1) was conducted 5 years after baseline and included 3,300 subjects. In 2007, the “Life-Events and Gene-Environment Interaction in Depression” (LEGEND) study was started based on SHIP (14). The lifetime diagnosis of MDD was assessed with the Munich-Composite International Diagnostic Interview (M-CIDI). The M-CIDI is a standardized fully structured instrument for assessing psychiatric disorders over the life span according to DSM-IV criteria. The computerized version of the interview was used by clinically experienced interviewers (psychologists) in a face-to-face situation. All interviewers had undergone intensive and continuous training in the diagnostic procedures. SHIP and LEGEND were approved by the local Institutional Review Board and conformed to the principles of the Declaration of Helsinki.

TwinGene

The TwinGene project, conducted between 2004 and 2008, is a population-based Swedish study of twins born between 1911 and 1958 drawn from the Swedish Twin Registry (15). All subjects in this investigation were independent (i.e., one twin selected per twin pair). The study participants previously participated in a telephone interview called Screening Across the Lifespan Twin Study (SALT). Data from the SALT study were used to identify MDD cases as either reporting antidepressant use or meeting DSM-IV criteria for MDD using the Composite International Diagnostic Interview Short Form (CIDI-SF) criteria. The study was approved by the local ethics committee at Karolinska Institutet and all participants gave informed consent.

Genetics of Recurrent Early-Onset Depression Phase II & Depression Genes and Networks

The second phase of GenRED was included as a replication sample (the first phase was a discovery sample). GenRED2 included new cases meeting the same criteria as in GenRED, plus new controls. Dr. Janet Sobell (University of Southern California) contributed 287 post-QC controls from the Mayo DNA Bank which consists of long-term, community medicine patients (Mayo Clinic, Rochester, MN) who were undergoing venipuncture for any reason. Consenting individuals ages 45 and above completed a brief demographic and psychiatric screening questionnaire. Extensive medical records were screened for evidence of psychiatric illness. Individuals were excluded if they were judged likely to have had a mood or psychotic disorder on the basis of a review of medical records, taking into account the recorded diagnoses and treatment in each case (including major disorders as well as possible proxies for a mood disorder in older nomenclature such as adjustment disorders, depression NOS, anxiety state, etc.). The final subset was selected to roughly match the proportions of self-reported ancestry in the GenRED 1+2 sample. Drs. Carlos Pato and James Knowles contributed 187 post-QC controls from the Genomic Psychiatry Consortium, an ongoing study of schizophrenia and bipolar disorder. Controls were recruited opportunistically in the Los Angeles area and screened with a self-report questionnaire. We selected individuals who reported European ancestry in all grandparents and answered negatively to five screening questions for lifetime bipolar disorder, five for lifetime schizophrenia, and four for lifetime MDD. Note that these 1,305 subjects were genotyped at the same lab (Centrillion Biosciences, Mountain View, CA) and with the same GWAS array (Illumina Omni1-Quad) as the Depression Genes and Networks sample (below) collected by a subset of the GenRED investigators, so that these samples were combined for the PGC replication analysis.

The Depression Genes and Networks (DGN) sample was genotyped and analyzed in combination with the GenRED2 sample (above). A survey research company (Knowledge Networks, Menlo Park, CA) recruited the 471 post-QC recurrent MDD cases and 470 never-depressed controls from participants in an online survey panel that is recruited on an ongoing basis using random digit dialing of nationally-representative US households. (Note that the same panel was used to recruit the Molecular Genetics of Schizophrenia control sample for the NIMH repository, but individuals who were invited to be screened for MGS and who were still members of the panel were not invited to be screened for DGN.) Online screening was carried out using the CIDI-SF depression and alcohol and substance dependence modules; prospective controls were selected who reported two or more episodes meeting criteria for MDD but denied lifetime substance dependence, while prospective controls denied ever having two or more weeks of depressed mood or anhedonia and two or more other MDD criteria outside of acute bereavement. These individuals were then interviewed (SCID) and individuals not meeting the initial eligibility criteria (based on final review by the site PI) were excluded. Note that these cases all reported recurrent MDD, but were not required to meet the additional criteria required for the GenRED project.

Danish Replication Sample

The Danish replication sample was made up of samples from 3 sources: additional RADIANT cases recruited in Denmark with genotype data available; a subset of Danish cases and screened controls recruited for the DEMO and PRISME studies genotyped specifically for these SNPs; and a cohort of unscreened controls from the Danish population already genotyped and imputed. As each sample individually was relatively small and minor allele frequencies were roughly in line across all samples, they were combined in their analysis.

The combined DEMO and PRISME samples came from 3 cohorts. The Denmark 1 cohort includes 162 cases with depression according to the ICD-10 diagnostic criteria. Diagnoses were obtained using a semi-structured diagnostic interview (Schedules for Clinical Assessment in Neuropsychiatry (SCAN version 2.1)). The subjects were all part of the Danish PRISME study and employees within large public service workplaces in a Danish county (16; 17). The Denmark 2 and 3 cohort includes 104 and 70 individuals from the Danish DEMO trial 1 and 2, respectively. The Denmark 2 cohort is described in detail by Krogh *et al.* (18; 19). All cases fulfilled the ICD-10 diagnostic criteria for depression and the diagnoses were obtained using the Major Depression Inventory (MDI) (20). The control sample consisted of 289 healthy, screened individuals with no diagnoses of affective disorders or anxiety disorders. The controls were all part of the Danish PRISME study. All cases and controls were Caucasian of origin and gave written informed consent. This study was approved by the Danish Data Protection Agency and the ethics

committees in Denmark. Genotyping for SNPs was done within this sample specifically for those requiring replication, as genome-wide data was not available. PCR reactions were composed of 10 ng DNA in a total volume of 9 µl. DNA amplification was performed as a multiplex reaction with either two (rs9323497 and rs7647854), two (rs10184299 and rs7950328) or four (rs625527, rs7807320, rs4379463 and rs1969420) markers simultaneously, except for rs1276324, which was amplified alone. Following PCR, primers and nucleotides were degraded by treatment with exonuclease and shrimp alkaline phosphatase and a primer extension reaction was performed according to the SNaPshot protocol using fluorescently labeled nucleotides (Applied Biosystems, Fostercity, USA). Genotyping was performed on an ABI 3130 Prism Genetic Analyzer, and the fluorescent peaks were analysed using Genemapper version 4.0 (Applied Biosystems, Fostercity, CA) and independently checked by two experienced investigators. Additional method conditions and primer sequences are available on request.

The 155 RADIANT cases were ascertained from two studies: DeNT, a retrospective study of depression in sibling pairs; and GENDEP, a pharmacogenetic study of antidepressant response. Both studies required moderate to severe depression as diagnosed by DSM-IV or ICD-10 criteria, with DeNT further requiring at least 2 episodes over their lifetime. All cases were recruited in Aarhus. Individuals were excluded if their genotypic data showed a missing rate >1%, abnormal heterozygosity, a sex assignment that conflicted with phenotypic data, if they were related (up to second degree) with other study members, or were of non-European ancestry. SNPs with a low minor allele frequency (<1%) or showing departure from Hardy–Weinberg equilibrium (p < 1E-5) were excluded. No imputation method was used. Full details of genotyping methods and quality control procedures has been published previously, with respect to the UK subset of the RADIANT studies (21).

The final cohort of Danish individuals were 883 controls from a schizophrenia GWAS conducted in Denmark on all individuals born in Denmark since 1981 having a schizophrenia diagnosis in the nationwide Danish Psychiatric Central Register as of 2006 (22). The controls were matched on sex, date of birth and age, and had no history of schizophrenia on the date of first diagnosis of schizophrenia of the case. By this procedure 915 controls were identified and subsequently dried blood spots from the individuals were obtained from the Danish Newborn Screening Biobank (DNSB) (23). Sufficient biological material was available for 899 controls. DNA was extracted from the dried blood spots using Extract-N-Amp Blood PCR kit (Sigma Aldrich) and subsequently whole-genome-amplified in triplicates using the RepliG kit (Qiagen) (24). The three separate reactions were pooled before genotyping, which was done using the Illumina Human 610-quad beadchip. Altogether 883 controls were successfully genotyped. SNPs with a deviation from Hardy-Weinberg equilibrium (p < 0.0001) and a MAF below 0.01 were

excluded as were SNPs and individuals with more than 2% missing data. Furthermore, test for relatedness, estimation of individual heterozygosity and test for non-random missingness of SNPs between cases and controls were conducted. One individual was excluded this way, leaving 882. Imputation was conducted using Impute v2.3.0 using standard settings and based on 1000 genome v3 and the strand file provided at the impute web site (25).

University of Münster Depression Cohorts

Data were pooled from the Münster Neuroimaging study and the Moodinflame study recruited from the same geographical area conducted at the Department of Psychiatry, University of Münster, Germany. From the Münster Neuroimaging Study, 516 healthy subjects, all adult, participated in the present study. All included subjects were thoroughly investigated by experienced psychologists and were free from any lifetime history of psychiatric disorders according to DSM-IV criteria (26), as ascertained by the SCID interview (27). Additional scales were employed (e.g., CTQ, LEQ, BDI). Exclusion criteria were scores ≥ 10 on the Beck Depression Inventory (BDI) (28), any neurological abnormalities, history of seizures, head trauma or unconsciousness, intake of any psychotropic medication, and the usual MRI contraindications. Depression cases were derived from two Münster Depression cohorts (Moodinflame and Münster Neuroimaging Study). As part of the Münster Neuroimaging Study of depression, 167 depressed adult subjects recruited since 2005 at the University of Münster, Germany. All included subjects were thoroughly investigated by experienced psychologists and major depressive disorder (MDD) was ascertained according to DSM-IV criteria (24), as diagnosed with the SCID interview (25). Patients were recruited from the inpatient service of the University of Münster's Department of Psychiatry. Exclusion criteria were any neurologic abnormalities; substance-related disorders; psychotic symptoms; a history of mania or hypomania; treatment with benzodiazepine; and previous electroconvulsive therapy; and the usual MRI-contraindications. Clinical assessment included a minimum of measures such as HAM-D-21, BDI, number of episodes of depression, age of onset, duration of illness, medication history, education age and sex. According to similar inclusion and exclusion criteria, a second cohort (Moodinflame) of 238 adult cases was also recruited at the University of Münster, Germany, as part of the Moodinflame study. Psychiatric diagnoses were ascertained using a structured clinical interview (SCID) (25). Clinical and pathological features were assessed in a standardized manner using various scales among which were Inventory of Depressive Symptoms - Clinician Rated (IDS-C), Young Mania Rating Scale (YMRS), Childhood Trauma Questionnaire (CTQ), HAM-D, medication and psychiatric history. In addition to age, sex, and education, clinical characteristics such as number of episodes of depression, age of onset of depression, duration of illness and medication history were assessed.

Genotyping was conducted blind to phenotypic data. The ABI PRSIM 7900HT Sequence Detection System (Applied Biosystems Inc., Foster City, CA, USA) was used to genotype using KASPtm genotyping assays (LGC Genomics Ltd, Hoddesdon, Herts, UK). Positive and negative controls were included four times on each 384-well plate run, and 100 samples were run in duplicate for each assay (with no conflicts). Deviation from Hardy Weinberg equilibrium was tested using chi-squared tests.

deCODE

Cases and controls were all Icelandic and were recruited from all over Iceland. For patients, diagnoses were assigned according to DSM-III or ICD-10 criteria. Controls were recruited as a part of various genetic programs at deCODE (excluding the schizophrenia, bipolar disorder, and anxiety projects) and were not screened for psychiatric disorders. Age at onset was obtained from hospital records and was defined as the age at first hospital admission. Approval for the study was granted by the National Bioethics Committee of Iceland and the Icelandic Data Protection Authority. Informed consent was obtained for all participants.

CONVERGE

CONVERGE (CONVERGE: China Oxford and VCU Experimental Research on Genetic Epidemiology) is a collaboration between the University of Oxford, Hua Shan Hospital at Fudan University, China, and Virginia Commonwealth University. Recurrent MD cases and controls were recruited from 53 provincial mental-health centers in China, were exclusively female and of Han Chinese ancestry. Critically, sampled controls have been carefully screened to ensure a low liability to depressive illness (>30 years old). Genetic material was extracted from saliva samples; sparse genome sequencing (~1.2X) was conducted for 12,000 cases and controls by Beijing Genomics Institute (BGI). Stringent quality control measures were implemented, with consideration to individual- and SNP-level metrics.

Generation Scotland

GS:SFHS consists of over 24,000 participants recruited at random from general medical practices across Scotland between 2006 and 2011. Probands (n = 7,953) were aged between 35 and 65 years and were registered with participating general medical practitioners. These individuals were not ascertained on the basis of having any particular disorder. Eligibility criteria specified that participants were over 18 years of age and had one first-degree relative also willing to participate. 20,223 individuals had phenotypic information for major depressive disorder (cases n = 2751, controls n = 17472; www.generationscotland.org/) (29; 30). Genome-wide SNP data were ascertained for 10,000 individuals, and after quality control, genotype data was available for 9863 participants. 18.1% of the genotyped

individuals had a diagnosis of single or recurrent episode depression. A shared family identity number was given to groups where each member was a first-degree relative of at least one other person. The current sample of unrelated individuals was ascertained by removing individuals with a shared family ID. Individuals with depression were preferentially selected from families. A diagnosis of MDD was made using the Structured Clinical Interview for DSM-IV Disorders (SCID); trained researchers administered the screening questions after a period of training and reliability assessment. Participants who screened positive (21.7%) were invited to continue the interview that focused on mood disorders, from which information on the presence or absence of a lifetime history of MDD, age of onset and number of depressive episodes was provided.

GERAD1 Consortium Study Description

Cases and elderly screened controls were recruited by the Medical Research Council (MRC) Genetic Resource for AD (Cardiff University; Institute of Psychiatry, London; Cambridge University; Trinity College Dublin), the Alzheimer's Research UK (ARUK) Collaboration (University of Nottingham; University of Manchester; University of Southampton; University of Bristol; Queen's University Belfast; the Oxford Project to Investigate Memory and Ageing (OPTIMA), Oxford University); Washington University, St Louis, United States; MRC PRION Unit, University College London; London and the South East Region AD project (LASER-AD), University College London; Competence Network of Dementia (CND) and Department of Psychiatry, University of Bonn, Germany; the National Institute of Mental Health (NIMH) AD Genetics Initiative. 6,129 population controls were drawn from large existing cohorts with available GWAS data, including the 1958 British Birth Cohort (1958BC) (http://www.b58cgene.sgul.ac.uk), the KORA F4 Study and the Heinz Nixdorf Recall Study. All AD cases met criteria for either probable (NINCDS-ADRDA, DSM-IV) or definite (CERAD) AD. All elderly controls were screened for dementia using the MMSE or ADAS-cog, were determined to be free from dementia at neuropathological examination or had a Braak score of 2.5 or lower. Genotypes from all cases and 4,617 controls were previously included in the AD GWAS by Harold and colleagues (4). Genotypes for the remaining 2,660 population controls were obtained from WTCCC2. Imputation of the dataset was performed using IMPUTE2 and the 1000 genomes (http://www.1000genomes.org/) Dec2010 reference panel (NCBI build 37.1). The imputed data were then analyzed using logistic regression including covariates for country of origin, sex, age and 3 principal components obtained with EIGENSTRAT software based on individual genotypes for the GERAD study participants.

GERAD1 Consortium Authorship

Denise Harold, Rebecca Sims, Amy Gerrish, Jade Chapman, Valentina Moskvina, Richard Abraham, Paul Hollingworth, Marian Hamshere, Jaspreet Singh Pahwa, Kimberley Dowzell, Amy Williams, Nicola Jones, Charlene Thomas, Alexandra Stretton, and Angharad Morgan, Medical Research Council (MRC) Centre for Neuropsychiatric Genetics and Genomics, Neurosciences and Mental Health Research Institute, Department of Psychological Medicine and Neurology, School of Medicine, Cardiff University, Cardiff, UK; Simon Lovestone, John Powell, Petroula Proitsi, and Michelle K. Lupton, Department of Neuroscience, Institute of Psychiatry, Kings College London, London, UK; Carol Brayne, Institute of Public Health, University of Cambridge, Cambridge, UK; David C. Rubinsztein, Cambridge Institute for Medical Research, University of Cambridge, Cambridge, UK; Michael Gill, Brian Lawlor, and Aoibhinn Lynch, Mercer's Institute for Research on Aging, St. James' Hospital and Trinity College, Dublin, Ireland; Kevin Morgan, and Kristelle Brown, Institute of Genetics, Queen's Medical Centre, University of Nottingham, Nottingham, UK; Peter Passmore, David Craig, Bernadette McGuinness, and Stephen Todd, Ageing Group, Centre for Public Health, School of Medicine, Dentistry and Biomedical Sciences, Queen's University Belfast, Belfast, UK; Clive Holmes, Division of Clinical Neurosciences, School of Medicine, University of Southampton, Southampton, UK; David Mann, Clinical Neuroscience Research Group, Greater Manchester Neurosciences Centre, University of Manchester, Salford, UK; A. David Smith, Oxford Project to Investigate Memory and Ageing (OPTIMA), University of Oxford, Level 4, John Radcliffe Hospital, Oxford, UK; Seth Love, and Patrick G. Kehoe, Dementia Research Group, University of Bristol Institute of Clinical Neurosciences, Frenchay Hospital, Bristol, UK; John Hardy, Department of Molecular Neuroscience and Reta Lilla Weston Laboratories, Institute of Neurology, London, UK; Simon Mead, MRC Prion Unit, Department of Neurodegenerative Disease, UCL Institute of Neurology, London, UK; Nick Fox, and Martin Rossor, Dementia Research Centre, Department of Neurodegenerative Diseases, University College, London, Institute of Neurology, London, UK; John Collinge, MRC Prion Unit, Department of Neurodegenerative Disease, UCL Institute of Neurology, London, UK; Wolfgang Maier, Frank Jessen, Reiner Heun, and Heike Kölsch, Department of Psychiatry, University of Bonn, Bonn, Germany; Britta Schürmann, Department of Psychiatry, and Institute for Molecular Psychiatry, University of Bonn, Bonn, Germany; Hendrik van den Bussche, Institute of Primary Medical Care, University Medical Center Hamburg Eppendorf, Germany; Isabella Heuser, Department of Psychiatry, Charité, Berlin, Germany; Johannes Kornhuber, Department of Psychiatry, University of Erlangen, Nürnberg, Germany; Jens Wiltfang, LVR Hospital Essen, Department of Psychiatry and Psychotherapy, University Duisburg-Essen, Germany; Martin Dichgans, Institute for Stroke and Dementia Research, and

Department of Neurology, Klinikum der Universität München, Munich, Germany; Lutz Frölich, Central Institute of Mental Health, Medical Faculty Mannheim, University of Heidelberg, Germany; Harald Hampel, Department of Psychiatry, Psychosomatic Medicine and Psychotherapy, Goethe University, Frankfurt, Germany; Michael Hüll, Centre for Geriatric Medicine and Section of Gerontopsychiatry and Neuropsychology, Medical School, University of Freiburg, Germany; Dan Rujescu, Alzheimer Memorial Center and Geriatric Psychiatry Branch, Department of Psychiatry, Ludwig-Maximilian University, Munich, Germany; Alison Goate, Departments of Psychiatry, Neurology and Genetics, Washington University School of Medicine, St. Louis, MO, USA; John S.K. Kauwe, Department of Biology, Brigham Young University, Provo, UT, USA; Carlos Cruchaga, Petra Nowotny, John C. Morris, and Kevin Mayo, Departments of Psychiatry, Neurology and Genetics, Washington University School of Medicine, St. Louis, MO, USA; Gill Livingston, Nicholas J. Bass, Hugh Gurling, and Andrew McQuillin, Department of Mental Health Sciences, University College London, London, UK; Rhian Gwilliam, and Panagiotis Deloukas, The Wellcome Trust Sanger Institute, Wellcome Trust Genome Campus, Hinxton, Cambridge, UK; Markus M. Nöthen, Department of Genomics, Life and Brain Center, University of Bonn, Bonn, Germany; Peter Holmans, Michael O'Donovan, Michael J. Owen, and Julie Williams, Medical Research Council (MRC) Centre for Neuropsychiatric Genetics and Genomics, Neurosciences and Mental Health Research Institute, Department of Psychological Medicine and Neurology, School of Medicine, Cardiff University, Cardiff, UK.

CONVERGE Consortium Authorship

Na Cai 1*, Tim Bigdeli 2*, Warren Kretzschmar 1*, Yihan Li 1*, Jieqin Liang 3, Li Song 3, Jingchu Hu 3, Qibin Li 3, Wei Jin 3, Zhenfei Hu 3, Guangbiao Wang 3, Linmao Wang 3, Puyi Qian 3, Yuan Liu 3, Tao Jiang 3, Yao Lu 3, Xiuqing Zhang 3, Ye Yin 3, Yingrui Li 3, Xun Xu 3, Xiangchao Gan 4, Mark Reimers 2, Todd Webb 2, Brien Riley 2, Silviu Bacanu 2, Roseann E Peterson 2, Yiping Chen 5, Hui Zhong 6, Zhengrong Liu 7, Gang Wang 8, Jing Sun 9, Hong Sang 10, Guoqing Jiang 11, Xiaoyan Zhou 11, Yi Li 12,13, Wei Zhang 14, Xueyi Wang 15, Xiang Fang 16, Runde Pan 17, Guodong Miao 18, Qiwen Zhang 19, Jian Hu 20, Fengyu Yu 21, Bo Du 22, Wenhua Sang 22, Keqing Li 22, Guibing Chen 23, Min Cai 24, Lijun Yang 25, Donglin Yang 26, Baowei Ha 27, Xiaohong Hong 28, Hong Deng 29, Gongying Li 30, Kan Li 31, Yan Song 32, Shugui Gao 33, Jinbei Zhang 34, Zhaoyu Gan 34, Huaqing Meng 35, Jiyang Pan 36, Chengge Gao 37, Kerang Zhang 38, Ning Sun 38, Youhui Li 39, Qihui Niu 39, Yutang Zhang 40, Tieqiao Liu 41, Chunmei Hu 42, Zhen Zhang 43, Luxian Lv 44, Jicheng Dong 45, Xiaoping Wang 46, Ming Tao 47, Xumei Wang 48, Jing Xia 48, Han Rong 49, Qiang He 50, Tiebang Liu 51, Guoping Huang 52, Qiyi Mei 53, Zhenming Shen 54,

Ying Liu 55, Jianhua Shen 56, Tian Tian 56, Xiaojuan Liu 57, Wenyuan Wu 58, Danhua Gu 59, Guangyi Fu 1, Yi Li 12,13, Jianguo Shi 60, Yunchun Chen 61, Jingfang Gao 62, Lanfen Liu 63, Lina Wang 63, Fuzhong Yang 64, Enzhao Cong 64, Jonathan Marchini 65,1, Huanming Yang 3, Jian Wang 3, Shenxun Shi 66,64, Richard Mott 1, Qi Xu 67 #, Jun Wang 3,68,69,70 #, Kenneth S Kendler 2 #, Jonathan Flint 71,1 #

* Denotes equal contribution; # Denotes corresponding author

1 Wellcome Trust Centre for Human Genetics, University of Oxford, Roosevelt Drive, Oxford, Oxfordshire, OX3 7BN, United Kingdom; 2 Virginia Institute for Psychiatric and Behavioral Genetics, Virginia Commonwealth University, Richmond, Virginia, 23298, USA; 3 BGI-Shenzhen, Floor 9 Complex Building, Beishan Industrial Zone, YantianDistrict, Shenzhen, Guangdong, 518083, China; 4 Department of Comparative Developmental Genetics, Max Planck Institute for Plant Breeding Research, Carl-von-Linne-Weg 10, Cologne, 50829, Germany; 5 CTSU, Richard Doll Building, University of Oxford, Old Road Campus, Oxford, Oxfordshire, OX3 7LF, United Kingdom; 6 Anhui Mental Health Center, No.316 Huangshan Road, Hefei, Anhui, 230000, China; 7 Anshan Psychiatric Rehabilitation Hospital, No.127 Shuangshan Road,Lishan District, Anshan, Liaoning, 114000, China; 8 Beijing Anding Hospital of Capital University of Medical Sciences, No.5 Ankang Hutong, Deshengmen wai, Xicheng District, Beijing, Beijing, 100000, China; 9 Brain Hospital of Nanjing Medical University, No.264 Guangzhou Road, Nanjing, Jiangsu, 210000, China; 10 Changchun Mental Hospital, No.4596 Beihuan Road, Changchun, Jilin, 130000, China; 11 Chongqing Mental Health Center, No.102 Jinzishan,Jiangbei District, Chongqing, 404100, China; 12 Dalian No.7 Hospital, No.179 Lingshui Road, Ganjingzi District, Dalian, Liaoning, 116000, China; 13 Wuhan Mental Health Center, No.70, Youyi Road, Wuhan, Hubei, 430000, China; 14 Daqing No.3 Hospital of Heilongjiang Province, No.54 Xitai Road, Ranghulu district, Daqing, Heilongjiang, 163000, China; 15 First Hospital of Hebei Medical University, No.89 Donggang Road,, Shijiazhuang, Hebei, 50000, China; 16 Fuzhou Psychiatric Hospital, No.451 South Erhuan Road,Cangshan District, Fuzhou, Fujian, 350000, China; 17 Guangxi Longquanshan Hospital, No.1 Jila Road, Yufeng District, Liuzhou, Guangxi Zhuangzu, 545000, China; 18 Guangzhou Brain Hospital, Guangzhou Psychiatric Hospital, No.36 Mingxin Road, Fangcun Avenue, Liwan District, Guangzhou, Guangdong, 510000, China; 19 Hainan Anning Hospital, No.10 East Nanhai Avenue, Haikou, Hainan, 570100, China; 20 Harbin Medical University, No.23 Youzheng street, Nangang District, Haerbin, Heilongjiang, 150000, China; 21 Harbin No.1 Special Hospital, No.217 Hongwei Road, Haerbin, Heilongjiang, 150000, China; 22 Hebei Mental Health Center, No.572 Dongfeng Road, Baoding, Hebei, 71000, China; 23 Huaian No.3 Hospital, No.272 West Huaihai Road, Huaian, Jiangsu, 223001, China; 24 Huzhou No.3 Hospital , No.255 Gongyuan

Road, Huzhou, Zhejiang, 313000, China; 25 Jilin Brain Hospital, No.98 West Zhongyang Road, Siping, Jilin, 136000, China; 26 Jining Psychiatric Hospital , North Dai Zhuang,Rencheng District, Jining, Shandong, 272000, China; 27 Liaocheng No. 4 Hospital, No.47 North Huayuan Road, Liaocheng, Shandong, 252000, China; 28 Mental Health Center of Shantou University, No.243 Daxue Road, Shantou, Guangdong, 515000, China; 29 Mental Health Center of West China Hospital of Sichuan University, No.28 South Dianxin Street, Wuhou District, Chengdu, Sichuan, 610000, China; 30 Mental Health Institute of Jining Medical College, Dai Zhuang, Bei Jiao, Jining, Shandong, 272000, China; 31 Mental Hospital of Jiangxi Province, No.43 Shangfang Road, Nanchang, Jiangxi, 330000, China; 32 Mudanjiang Psychiatric Hospital of Heilongjiang Province, Xinglong, Mudanjiang, Heilongjiang, 157000, China; 33 Ningbo Kang Ning Hospital, No.1 Zhuangyu Road ,Zhenhai District, Ningbo, Zhejiang, 315000, China; 34 No. 3 Hospital of Sun Yat-sen University, No.600 Tianhe Road, Tianhe District, Guangzhou, Guangdong, 510630, China; 35 No.1 Hospital of Chongqing Medical University, No.1 Youyi Road,Yuanjiagang,Yuzhong District, Chongqing, Chongqing, 400016, China; 36 No.1 Hospital of Jinan University, No.613 West Huangpu Avenue, Guangzhou, Guangdong, 510000, China; 37 No.1 Hospital of Medical College of Xian Jiaotong University, No. 277 West Yan Ta Road, Xian, Shaan Xi , 710061, China; 38 No.1 Hospital of Shanxi Medical University, No.85 South Jiefang Road, Taiyuan, Shanxi, 30000, China; 39 No.1 Hospital of Zhengzhou University, No.1 East Jianshe Road, Zhengzhou, Henan, 450000, China; 40 No.2 Hospital of Lanzhou University, No.82, Cuiyingmen, Lanzhou, Gansu, 730000, China; 41 No.2 Xiangya Hospital of Zhongnan University, No.139 Middle Renmin Road,Furong District, Changsha, Hunan, 410000, China; 42 No.3 Hospital of Heilongjiang Province, No.135 Jiaotong Road, Beian, Heilongjiang, 164000, China; 43 No.4 Hospital of Jiangsu University, No.246 Nanmen Street, Zhenjiang, Jiangsu, 212000, China; 44 Psychiatric Hospital of Henan Province, No.388 Middle Jianshe Road, Xinxiang, Henan, 453000, China; 45 Qingdao Mental Health Center, No.299 Nanjing Road, Shibei District, Qingdao, Shandong, 266000, China; 46 Renmin Hospital of Wuhan University, No.238 Jiefang Road, Wuchang District, Wuhan, Hubei, 430000, China; 47 Second Affiliated Hospital of Zhejiang Chinese Medical University, No.318 Chaowang Road, Hangzhou, Zhejiang, 310000, China; 48 ShengJing Hospital of China Medical University, No.36 Sanhao Street, Heping District, Shenyang, Liaoning, 110001, China; 49 Shenzhen Key Lab for Psychological Healthcare, Kangning Hospital, No.1080, Cuizhu Street, Luohu District, Shenzhen, Guangdong, 518000, China; 50 Department of General Internal Medicine, Kanazawa Medical University, Kahoku, Ishikawa, 920-0293, Japan; 51 Shenzhen Key Lab for Psychological Healthcare;Shenzhen Kangning Hospital, No.1080, Cuizhu Street, Luohu District , Shenzhen, Guangdong, 518000, China; 52 Sichuan Mental Health Center, No.190, East Jiannan Road, Mianyang, Sichuan, 621000, China; 53 Suzhou

Guangji Hospital, No.286, Guangji Road, Suzhou, Jiangsu, 215000, China; 54 Tangshan No.5 Hospital, No.57 West Nanxin Road, Lunan District, Tangshan, Hebei, 63000, China; 55 The First Hospital of China Medical University, No.155 North Nanjing Street, Heping District, Shenyang, Liaoning, 110001, China; 56 Tianjin Anding Hospital, No.13 Liulin Road, Hexi District, Tianjin, 300000, China; 57 Tianjin First Center Hospital, No.55 Xuetang Street, Xinkai Road, Hedong District, Tianjin, 300000, China; 58 Tongji University Hospital, No.389 Xinchun Road, Shanghai, 200000, China; 59 Weihai Mental Health Center, Qilu Avenue, ETDZ, Weihai, Shandong, 264200, China; 60 Xian Mental Health Center, No.15 Yanyin Road, New Qujiang District, Xian, Shaanxi, 710000, China; 61 Xijing Hospital of No.4 Military Medical University, No.17 West Changle Road, Xian, Shaanxi, 710000, China; 62 Zhejiang Traditional Chinese Medical Hospital, No.54 Youdian Road, Hangzhou, Zhejiang, 310000, China; 63 Shandong Mental Health Center, No.49 East Wenhua Road, Jinan, Shandong, 250000, China; 64 Shanghai Jiao Tong University School of Medicine, Shanghai Mental Health Centre, No. 600 Wan Ping Nan Road, Shanghai, 200030, China; 65 Department of Statistics, University of Oxford, Oxford, Oxfordshire, OX1 3TG, United Kingdom; 66 Fudan University affiliated Huashan Hospital, No. 12 Wulumuqi Zhong Road, Shanghai, 200040, China; 67 National Laboratory of Medical Molecular Biology, Institute of Basic Medical Sciences & Neuroscience Center, Chinese Academy of Medical Sciences and Peking Union Medical College, Beijing, 10005, China; 68 Department of Biology, University of Copenhagen, Ole Maal Oes Vej 5, Copenhagen, 2200, Denmark; 69 Macau University of Science and Technology, Avenida Wai long, Taipa, Macau 999078, China, Taipa, Macau, 999078, China; 70 Princess Al Jawhara Center of Excellence in the Research of Hereditary Disorders, King Abdulaziz University, Jeddah, 21589, Saudi Arabia; 71 East China Normal University, 3663 North Zhongshan Road, Shanghai, 200062, China

CARDIoGRAM Consortium Authorship

Heribert Schunkert[1], Inke R. König[2], Sekar Kathiresan[3,4,5], Muredach P. Reilly[6], Themistocles L. Assimes[7], Hilma Holm[8], Michael Preuss[1,2], Alexandre F. R. Stewart[9], Maja Barbalic[10], Christian Gieger[11], Devin Absher[12], Zouhair Aherrahrou[1], Hooman Allayee[13], David Altshuler[5,14], Sonia S. Anand[15], Karl Andersen[16,17], Jeffrey L. Anderson[18], Diego Ardissino[19], Stephen G. Ball[20,21], Anthony J. Balmforth[22], Timothy A. Barnes[23], Diane M. Becker[24], Lewis C. Becker[24], Klaus Berger[25], Joshua C. Bis[26], S. Matthijs Boekholdt[27,28], Eric Boerwinkle[10], Peter S. Braund[23], Morris J. Brown[29], Mary Susan Burnett[30], Ian Buysschaert[31,32], Cardiogenics, John F. Carlquist[18], Li Chen[33], Sven Cichon[34,35,36], Veryan Codd[23], Robert W. Davies[37], George Dedoussis[38], Abbas Dehghan[39,40], Serkalem Demissie[41,42], Joseph M. Devaney[30], Ron Do[43], Angela Doering[11], Sandra Eifert[44], Nour Eddine El Mokhtari[45], Stephen G. Ellis[46], Roberto Elosua[47],

James C. Engert[43,48], Stephen E. Epstein[30], Ulf de Faire[49,50], Marcus Fischer[51], Aaron R. Folsom[52], Jennifer Freyer[1], Bruna Gigante[49,50], Domenico Girelli[53], Solveig Gretarsdottir[8], Vilmundur Gudnason[17,54], Jeffrey R. Gulcher[8], Eran Halperin[55,56,57], Naomi Hammond[58], Stanley L. Hazen[59], Albert Hofman[39], Benjamin D. Horne[18], Thomas Illig[11], Carlos Iribarren[60], Gregory T. Jones[61], J.Wouter Jukema[62,63], Michael A. Kaiser[23], Lee M. Kaplan[64], John J.P. Kastelein[65], Kay-Tee Khaw[66], Joshua W. Knowles[7], Genovefa Kolovou[67], Augustine Kong[8], Reijo Laaksonen[68], Diether Lambrechts[32], Karin Leander[49], Guillaume Lettre[69,70], Mingyao Li[71], Wolfgang Lieb[1], Patrick Linsel-Nitschke[1], Christina Loley[1,2], Andrew J. Lotery[72,73], Pier M. Mannucci[74], Seraya Maouche[1], Nicola Martinelli[53], Pascal P. McKeown[75], Christa Meisinger[11], Thomas Meitinger[76,77], Olle Melander[78], Pier Angelica Merlini[79], Vincent Mooser[80], Thomas Morgan[81], Thomas W. Mühleisen[34,35], Joseph B. Muhlestein[18], Thomas Münzel[82], Kiran Musunuru[3,4,5], Janja Nahrstaedt[1,2], Christopher P. Nelson[22], Markus M. Nöthen[34,35], Oliviero Olivieri[53], Riyaz S. Patel[83,84], Chris C. Patterson[75], Annette Peters[11], Flora Peyvandi[85], Liming Qu[71], Arshed A. Quyyumi[83], Daniel J. Rader[6,86], Loukianos S. Rallidis[87], Catherine Rice[58], Frits R. Rosendaal[88,89,90], Diana Rubin[91], Veikko Salomaa[92], M. Lourdes Sampietro[93], Manj S. Sandhu[94,95], Eric Schadt[96,97], Arne Schäfer[98], Arne Schillert[2], Stefan Schreiber[98], Jürgen Schrezenmeir[99,100], Stephen M. Schwartz[26], David S. Siscovick[26], Mohan Sivananthan[101], Suthesh Sivapalaratnam[27], Albert Smith[17,54], Tamara B. Smith[102], Jaapjan D. Snoep[88], Nicole Soranzo[58], John A. Spertus[103], Klaus Stark[51], Kathy Stirrups[58], Monika Stoll[104], W. H. Wilson Tang[46], Stephanie Tennstedt[1], Gudmundur Thorgeirsson[16,17], Gudmar Thorleifsson[8], Maciej Tomaszewski[23,105], Andre G. Uitterlinden[39,106,40], Andre M. van Rij[61], Benjamin F. Voight[4,5,107], Nick J. Wareham[108], George A. Wells[37], H.-Erich Wichmann[11,44,109], Philipp S. Wild[82], Christina Willenborg[1,2], Jaqueline C. M. Witteman[39,40], Benjamin J. Wright[110], Shu Ye[111], Tanja Zeller[82], Andreas Ziegler[2], Francois Cambien[112], Alison H. Goodall[23,105], L. Adrienne Cupples[41,42], Thomas Quertermous[7], Winfried März[113,114,115], Christian Hengstenberg[51], Stefan Blankenberg[82], Willem H. Ouwehand[116,58], Alistair S. Hall[21], Panos Deloukas[58], John R. Thompson[117], Kari Stefansson[8,17], Robert Roberts[9], Unnur Thorsteinsdottir[8,17], Christopher J. O'Donnell[42], Ruth McPherson[9,118], Jeanette Erdmann[1], and Nilesh J. Samani[23,105.]

1 Universität zu Lübeck, Medizinische Klinik II, Lübeck, Germany; 2 Institut für Medizinische Biometrie und Statistik, Universität zu Lübeck, Lübeck, Germany; 3 Cardiovascular Research Center and Cardiology Division, Massachusetts General Hospital, Boston, MA, USA; 4 Center for Human Genetic Research, Massachusetts General Hospital, Boston, MA, USA; 5 Program in Medical and Population Genetics, Broad Institute of MIT and Harvard, Cambridge, MA, USA; 6 The Cardiovascular Institute, University of Pennsylvania, Philadelphia, Pennsylvania 19104, USA; 7 Department of Medicine, Stanford University

School of Medicine, Stanford, CA, USA; 8 deCODE Genetics, 101 Reykjavik, Iceland; 9 The John & Jennifer Ruddy Canadian Cardiovascular Genetics Centre, University of Ottawa Heart Institute, Ottawa, Canada; 10 University of Texas Health Science Center, Human Genetics Center, Houston, TX, USA; 11 Institute of Epidemiology, Helmholtz Zentrum München, German Research Center for Environmental Health, Neuherberg, Germany; 12 Hudson Alpha Institute, Huntsville, Alabama, USA; 13 Department of Preventive Medicine University of Southern California Los Angeles, CA USA; 14 Department of Molecular Biology and Center for Human Genetic Research, Massachusetts General Hospital, Harvard Medical School, Boston, USA; 15 Population Health Research Institute, Hamiliton Health Sciences and McMaster University, Hamilton, Ontario, Canada; 16 Department of Medicine, Landspitali University Hospital, 101 Reykjavik, Iceland; 17 University of Iceland, Faculty of Medicine, 101 Reykjavik, Iceland; 18 Cardiovascular Department, Intermountain Medical Center; Cardiology Division, University of Utah. Salt Lake City, UT, USA; 19 Division of Cardiology, Azienda Ospedaliero-Universitaria di Parma, Parma, Italy 20LIGHT Research Institute, Faculty of Medicine and Health, University of Leeds, Leeds, UK 21Division of Cardiovascular and Neuronal Remodelling, Multidisciplinary Cardiovascular Research Centre, Leeds Institute of Genetics, Health and Therapeutics, University of Leeds, Leeds, UK 22Division of Cardiovascular and Diabetes Research, Multidisciplinary Cardiovascular Research Centre, Leeds Institute of Genetics, Health and Therapeutics, University of Leeds, Leeds, LS2 9JT, UK 23Department of Cardiovascular Sciences, University of Leicester, Clinical Sciences Wing, Glenfield Hospital, Leicester, LE3 9QP, UK 24The Johns Hopkins University School of Medicine, Division of General Internal Medicine, Baltimore, MD 21287 25Institute of Epidemiology and Social Medicine, University of Münster, Germany 26Cardiovascular Health Resarch Unit and Department of Medicine, University of Washington, Seattle, WA USA 27Department of Vascular Medicine, Academic Medical Center, Amsterdam, The Netherlands 28Department of Cardiology, Academic Medical Center, Amsterdam, The Netherlands 29Clinical Pharmacology Unit, University of Cambridge, Cambridge, UK 30Cardiovascular Research Institute, Medstar Health Research Institute, Washington Hospital Center, Washington, DC 20010, USA 31Department of Cardiology, University Hospital Gasthuisberg, Leuven, Belgium 32Vesalius Research Center, VIB-KULeuven, Leuven, Belgium 33Cardiovascular Research Methods Centre, University of Ottawa Heart Institute, 40 Ruskin Street, Ottawa, Ontario, Canada, K1Y 4W7 34Institute of Human Genetics, University of Bonn, Bonn, Germany 35Department of Genomics, Life & Brain Center, University of Bonn, Bonn, Germany 36Institute of Neuroscience and Medicine (INM-1), Research Center Juelich, Juelich, Germany 37The Cardiovascular Research Methods, University of Ottawa Heart Institute, Ottawa, Ontario, Canada 38Department of Dietetics-Nutrition, Harokopio University, 17671 Athens,

Greece 39Department of Epidemiology, Erasmus Medical Center, Rotterdam, The Netherlands 40Member of Netherlands Consortium for Healthy Aging (NCHA) sponsored by Netherlands Genomics Initiative (NGI), Leiden, The Netherlands 41Department of Biostatistics, Boston University School of Public Health, Boston, MA USA 42National Heart, Lung and Blood Institute's Framingham Heart Study, Framingham, MA, USA 43Department of Human Genetics, McGill University, Montreal, Canada 44Klinikum Grosshadern, Munich, Germany 45Klinik für Innere Medizin, Kreiskrankenhaus Rendsburg, Rendsburg, Germany 46Department Cardiovascular Medicine, Cleveland Clinic, Cleveland, USA 47Cardiovascular Epidemiology and Genetics Group, Institut Municipal d'Investigació Mèdica, Barcelona; Ciber Epidemiología y Salud Pública (CIBERSP), Spain 48Department of Medicine, McGill University, Montreal, Canada 49Division of Cardiovascular Epidemiology, Institute of Environmental Medicine, Karolinska Institutet, Stockholm, Sweden 50Department of Cardiology, Karolinska University Hospital, Stockholm, Sweden 51Klinik und Poliklinik für Innere Medizin II, Universität Regensburg, Regensburg, Germany 52University of Minnesota School of Public Health, Division of Epidemiology and Community Health, School of Public Health (A.R.F.), Minneapolis, Minn.; USA 53Department of Medicine, University of Verona, Verona, Italy 54Icelandic Heart Association, Kopavogur Iceland 55The Blavatnik School of Computer Science , Tel-Aviv University, Tel-Aviv, Israel 56Department of Molecular Microbiology and Biotechnology, Tel-Aviv University, Tel-Aviv, Israel 57International Computer Science Institute, Berkeley, CA, USA 58Wellcome Trust Sanger Institute, Wellcome Trust Genome Campus, Hinxton, UK 59Lerner Research Institute, Cleveland Clinic, Cleveland, USA 60Division of Research, Kaiser Permanente of Northern California, Oakland, California, USA 61Surgery Department, Dunedin School of Medicine, University of Otago, New Zealand 62Department of Cardiology C5-P, Leiden University Medical Center, Leiden, The Netherlands 63Durrer Center for Cardiogenetic Research, Amsterdam, The Netherlands 64Massachusetts General Hospital, Boston MA, USA 65Dept. Vascular Medicine, Academic Medical Center, University of Amsterdam, Amsterdam, The Netherlands 66Department of Public Health and Primary Care, Strangeways Research Laboratory, University of Cambridge, Cambridge, UK 671st Cardiology Department, Onassis Cardiac Surgery Center, 356 Sygrou Avenue, 17674 Athens, Greece 68Science Center, Tampere University Hospital, Tampere, Finland 69Montreal Heart Institute, Montréal, Québec, H1T 1C8, Canada 70Département de Médecine, Université de Montréal, C.P. 6128, succursale Centre-ville, Montréal, Québec, H3C 3J7, Canada 71Biostatistics and Epidemiology, University of Pennsylvania, Philadelphia, PA, USA 72Clinical Neurosciences Division, School of Medicine, University of Southampton, Southampton, UK 73Southampton Eye Unit, Southampton General Hospital, Southampton, UK 74Scientific Direction, IRCCS Fondazione Cà Granda, Ospedale Maggiore Policlinico,

Milano, Italy 75Centre for Public Health, Queen's University Belfast, Institute of Clinical Science, Belfast, Northern Ireland, UK 76Institute of Human Genetics, Helmholtz Zentrum München, Deutsches Forschungszentrum für Umwelt und Gesundheit, Neuherberg, Germany 77Institute of Human Genetics, Technische Universität München, Klinikum rechts der Isar, Munich, Germany 78Department of Clinical Sciences, Hypertension and Cardiovascular Diseases, Scania University Hospital, Lund University, Malmö, Sweden 79Division of Cardiology, Azienda Ospedaliera Niguarda Ca'Granda, Milan, Italy 80Genetics Division and Drug Discovery, GlaxoSmithKline, King of Prussia, Pennsylvania 19406, USA 81Department of Pediatrics, Vanderbilt University School of Medicine, Nashville 822. Medizinische Klinik und Poliklinik, Universitätsmedizin Mainz, JohannesGutenberg Universität Mainz, Germany 83Emory University School of Medicine, Atlanta GA, USA 84Cardiff University, Cardiff, Wales, UK CF10 3XQ 85A. Bianchi Bonomi Hemophilia and Thrombosis Center, Department of Medicine and Medical Specialties, Fondazione IRCCS Ca Granda, Ospedale Maggiore Policlinico, Università degli Studi di Milano and Luigi Villa Foundation, Milan, Italy 86The Institute for Translational Medicine and Therapeutics, School of Medicine, University of Pennsylvania, Philadelphia, PA, USA 87Second Department of Cardiology, Attikon Hospital, School of Medicine, University of Athens, Athens, Greece 88Department of Clinical Epidemiology, Leiden University Medical Center, Leiden, The Netherlands 89Department of Thrombosis and Haemostasis, Leiden University Medical Center, Leiden, The Netherlands 90Einthoven Laboratory for Experimental Vascular Medicine, Leiden University Medical Center, Leiden, The Netherlands 91Medizinische Klinik I, Universitätsklinikum Schleswig-Holstein, Campus Kiel, Kiel, Germany 92Chronic Disease Epidemiology and Prevention Unit, Department of Chronic Disease Prevention, National Institute for Health and Welfare, Helsinki, Finland 93Department of Human Genetics and Cardiology, Leiden University Medical Center, Leiden, the Netherlands 94Manjinder S Sandhu, Genetic Epidemiology Group, Wellcome Trust Sanger Institute, Cambridge, UK 95Department of Public Health & Primary Care, Strangeways Research Laboratory, University of Cambridge, UK 96Pacific Biosciences, 1505 Adams Drive, Menlo Park, CA 94025 97Sage Bionetworks, Palo Alto, CA 94301 98Institut für Klinische Molekularbiologie, Christian-Albrechts Universität, Kiel, Germany 99Institute of Physiology and Biochemistry of Nutrition, Max Rubner-Institute, Kiel, Germany 100Clinical Research Center Kiel, Kiel Innovation and Technology Center, Kiel, Germany 101Cardiology Division, Leeds Teaching Hospitals NHS Trust, Leeds, UK 102Laboratory of Epidemiology, Demography, and Biometry, Intramural Research Program, National Institute on Aging, National Institutes of Health, Bethesda MD, USA 103Mid America Heart Institute and University of Missouri-Kansas City, Kansas City 104LeibnizInstitute for Arteriosclerosis Research, University of Münster, Münster, Germany 105Leicester National Institute for Health Research Biomedical Research

Unit in Cardiovascular Disease, Glenfield Hospital, Leicester, LE3 9QP, UK 106Department of Internal Medicine, Erasmus Medical Center, Rotterdam, The Netherlands 107Department of Medicine, Harvard Medical School, Boston, MA, USA 108MRC Epidemiology Unit, Institute of Metabolic Science, Addenbrooke's Hospital, Cambridge, UK 109Institute of Medical Information Science, Biometry and Epidemiology, Ludwig-Maximilians-Universität München, Germany 110Department of Cardiovascular Surgery, University of Leicester, Leicester, UK 111William Harvey Research Institute, Barts and The London School of Medicine and Dentistry, Queen Mary University of London, London, UK 112INSERM UMRS 937, Pierre and Marie Curie University, UPMC-Paris 6, Faculté de Médecine Pierre et Marie Curie, Paris, France 113Synlab Center of Laboratory Diagnostics Heidelberg, Heidelberg, Germany 114Clinical Institute of Medical and Chemical Laboratory Diagnostics, Medical University of Graz, Graz, Austria 115Institute of Public Health, Social and Preventive Medicine, Medical Faculty Manneim, University of Heidelberg, Germany 116Department of Haematology, University of Cambridge and NHS Blood and Transplant, Cambridge, UK 117Department of Health Sciences, University of Leicester, Leicester, UK 118Atherogenomics Laboratory, University of Ottawa Heart Institute, Ottawa, Canada

Supplemental References

1. Macgregor S, Craddock N, Holmans PA. (2006): Use of phenotypic covariates in association analysis by sequential addition of cases. *European journal of human genetics : EJHG* 14: 529-534.
2. Sklar P, Ripke S, Scott LJ, Andreassen OA, Cichon S, Craddock N, *et al.* (2011): Large-scale genome-wide association analysis of bipolar disorder identifies a new susceptibility locus near ODZ4. *Nat Genet* 43: 977-U162.
3. Ripke S, Sanders AR, Kendler KS, Levinson DF, Sklar P, Holmans PA, *et al.* (2011): Genome-wide association study identifies five new schizophrenia loci. *Nat Genet* 43: 969-U977.
4. Harold D, Abraham R, Hollingworth P, Sims R, Gerrish A, Hamshere ML, *et al.* (2009): Genome-wide association study identifies variants at CLU and PICALM associated with Alzheimer's disease. *Nat Genet* 41: 1088-1093.
5. Schunkert H, Konig IR, Kathiresan S, Reilly MP, Assimes TL, Holm H, *et al.* (2011): Large-scale association analysis identifies 13 new susceptibility loci for coronary artery disease. *Nat Genet* 43: 333-338.
6. Yang JA, Lee SH, Goddard ME, Visscher PM. (2011): GCTA: A Tool for Genome-wide Complex Trait Analysis. *Am J Hum Genet* 88: 76-82.
7. Lee SH, Decandia TR, Ripke S, Yang J, Sullivan PF, Goddard ME, *et al.* (2012): Estimating the proportion of variation in susceptibility to schizophrenia captured by common SNPs (vol 44, pg 247, 2012). *Nat Genet* 44: 831-831.

8. Kessler RC, Berglund P, Demler O, Jin R, Merikangas KR, Walters EE. (2005): Lifetime prevalence and age-of-onset distributions of DSM-IV disorders in the national comorbidity survey replication.(vol 62, pg 593, 2005). *Arch Gen Psychiat* 62: 768-768.
9. Firmann M, Mayor V, Vidal PM, Bochud M, Pecoud A, Hayoz D*, et al.* (2008): The CoLaus study: a population-based study to investigate the epidemiology and genetic determinants of cardiovascular risk factors and metabolic syndrome. *BMC Cardiovasc Disord* 8: 6.
10. Preisig M, Waeber G, Vollenweider P, Bovet P, Rothen S, Vandeleur C*, et al.* (2009): The PsyCoLaus study: methodology and characteristics of the sample of a population-based survey on psychiatric disorders and their association with genetic and cardiovascular risk factors. *Bmc Psychiatry* 9: 9.
11. Nurnberger JI, Blehar MC, Kaufmann CA, York-Cooler C, Simpson SG, Harkavy-Friedman J*, et al.* (1994): Diagnostic Interview for Genetic Studies: rationale, unique features, and training. *Archives of General Psychiatry* 51: 849-859.
12. Grabe HJ, Lange M, Wolff B, Volzke H, Lucht M, Freyberger HJ*, et al.* (2005): Mental and physical distress is modulated by a polymorphism in the 5-HT transporter gene interacting with social stressors and chronic disease burden. *Mol Psychiatry* 10: 220-224.
13. Henquet C, Krabbendam L, Spauwen J, Kaplan C, Lieb R, Wittchen HU*, et al.* (2005): Prospective cohort study of cannabis use, predisposition for psychosis, and psychotic symptoms in young people. *BMJ* 330: 11.
14. Volzke H, Alte D, Schmidt CO, Radke D, Lorbeer R, Friedrich N*, et al.* (2011): Cohort profile: the study of health in Pomerania. *International journal of epidemiology* 40: 294-307.
15. Lichtenstein P, Sullivan PF, Cnattingius S, Gatz M, Johansson S, Carlstrom E*, et al.* (2006): The Swedish Twin Registry in the third millennium: an update. *Twin Res Hum Genet* 9: 875-882.
16. Kolstad HA, Hansen AM, Kaergaard A, Thomsen JF, Kaerlev L, Mikkelsen S*, et al.* (2011): Job strain and the risk of depression: is reporting biased? *Am J Epidemiol* 173: 94-102.
17. Elfving B, Buttenschon HN, Foldager L, Poulsen PH, Andersen JH, Grynderup MB*, et al.* (2012): Depression, the Val66Met polymorphism, age, and gender influence the serum BDNF level. *Journal of psychiatric research* 46: 1118-1125.
18. Krogh J, Petersen L, Timmermann M, Saltin B, Nordentoft M. (2007): Design paper: the DEMO trial: a randomized, parallel-group, observer-blinded clinical trial of aerobic versus non-aerobic versus relaxation training for patients with light to moderate depression. *Contemp Clin Trials* 28: 79-89.
19. Krogh J, Videbech P, Thomsen C, Gluud C, Nordentoft M. (2012): DEMO-II trial. Aerobic exercise versus stretching exercise in patients with major depression-a randomised clinical trial. *PLoS One* 7: e48316.
20. Bech P, Rasmussen NA, Olsen LR, Noerholm V, Abildgaard W. (2001): The sensitivity and specificity of the Major Depression Inventory, using the Present State Examination as the index of diagnostic validity. *J Affect Disord* 66: 159-164.
21. Lewis CM, Ng MY, Butler AW, Cohen-Woods S, Uher R, Pirlo K*, et al.* (2010): Genome-Wide Association Study of Major Recurrent Depression in the UK Population. *American Journal of Psychiatry* 167: 949-957.
22. Borglum AD, Demontis D, Grove J, Pallesen J, Hollegaard MV, Pedersen CB*, et al.* (2013): Genome-wide study of association and interaction with maternal cytomegalovirus infection suggests new schizophrenia loci. *Mol Psychiatry*.
23. Henquet C, Di Forti M, Morrison P, Kuepper R, Murray RM. (2008): Gene-environment interplay between cannabis and psychosis. *Schizophr Bull* 34: 1111-1121.
24. Agrawal A, Lynskey MT. (2006): The genetic epidemiology of cannabis use, abuse and dependence. *Addiction* 101: 801-812.

25. Verweij KJ, Vinkhuyzen AA, Benyamin B, Lynskey MT, Quaye L, Agrawal A, *et al.* (2013): The genetic aetiology of cannabis use initiation: a meta-analysis of genome-wide association studies and a SNP-based heritability estimation. *Addict Biol* 18: 846-850.
26. American Psychiatric Association, 1994. Diagnostic and Statistical Manual of Mental Disorders, 4th edn. American Psychiatric Association, Washington, DC.
27. Wittchen H-U, Wunderlich U, Gruschwitz S, Zaudig M, 1997. SKID-I. Strukturiertes Klinisches Interview für DSM-IV. . Hogrefe, Göttingen, Germany.
28. Beck AT, Steer RA, 1987. Beck Depression Inventory: manual. Psychological Corporation Harcourt Brace Jovanovich, San Antonio, U.S.A.
29. Smith BH, Campbell H, Blackwood D, Connell J, Connor M, Deary IJ, *et al.* (2006): Generation Scotland: the Scottish Family Health Study; a new resource for researching genes and heritability. *BMC Med Genet* 7: 74.
30. Smith BH, Campbell A, Linksted P, Fitzpatrick B, Jackson C, Kerr SM, *et al.* (2013): Cohort Profile: Generation Scotland: Scottish Family Health Study (GS:SFHS). The study, its participants and their potential for genetic research on health and illness. *Int J Epidemiol* 42: 689-700.
